# Yeast mannan rich fraction positively influences microbiome uniformity, productivity associated taxa, and lay performance

**DOI:** 10.1186/s42523-024-00295-7

**Published:** 2024-03-04

**Authors:** Robert J. Leigh, Aoife Corrigan, Richard A. Murphy, Jules Taylor-Pickard, Colm A. Moran, Fiona Walsh

**Affiliations:** 1 Department of Biology, Maynooth University, Maynooth, Co. Kildare Ireland; 2 Alltech Bioscience Centre, Dunboyne, Co. Meath Ireland; 3 Alltech (UK) Ltd., Stamford, PE9 1TZ United Kingdom; 4 Alltech SARL, Rue Charles Amand, 14500 Vire, France

## Abstract

**Background:**

Alternatives to antibiotic as growth promoters in agriculture, such as supplemental prebiotics, are required to maintain healthy and high performing animals without directly contributing to antimicrobial resistance bioburden. While the gut microbiota of broiler hens has been well established and successfully correlated to performance, to our knowledge, a study has yet to be completed on the effect of prebiotic supplementation on correlating the mature laying hen productivity and microbiota. This study focused on establishing the impact of a yeast derived prebiotic, mannan rich fraction (MRF), on the cecal microbiota of late laying hens. This study benefitted from large sample sizes so intra- and intergroup variation effects could be statistically accounted for.

**Results:**

Taxonomic richness was significantly greater at all taxonomic ranks and taxonomic evenness was significantly lower for all taxonomic ranks in MRF-supplemented birds (*P* < 0.005). Use of principal coordinate analyses and principal component analyses found significant variation between treatment groups. When assessed for compositional uniformity (an indicator of flock health), microbiota in MRF-supplemented birds was more uniform than control birds at the species level. From a food safety and animal welfare perspective, *Campylobacter jejuni* was significantly lower in abundance in MRF-supplemented birds. In this study, species associated with high weight gain (an anticorrelator of performance in laying hens) were significantly lower in abundance in laying hens while health-correlated butyrate and propionate producing species were significantly greater in abundance in MRF-supplemented birds.

**Conclusions:**

The use of prebiotics may be a key factor in controlling the microbiota balance limiting agri-food chain pathogen persistence and in promoting uniformity. In previous studies, increased α- and β-diversity indices were determinants of pathogen mitigation and performance. MRF-supplemented birds in this study established greater α- and β-diversity indices in post-peak laying hens, greater compositional uniformity across samples, a lower pathogenic bioburden and a greater abundance of correlators of performance.

**Supplementary Information:**

The online version contains supplementary material available at 10.1186/s42523-024-00295-7.

## Introduction

Microbiota alteration and microbiome augmentation has been particularly successful in promoting flock health in both broilers and laying hens [19, 22, 53, 69]. As the hen gut microbiome responds well to dietary factors, this provides a manipulation susceptible and cost-effective avenue for more sustainable meat and egg production [2, 13, 90].

Like all Vertebrata, the gastrointestinal tract of hens (*Gallus gallus* subsp. *domesticus*) is richly colonised by dynamic and interactive microbial communities that respond to biotic and abiotic factors such as diet, stress, and the circadian cycle [75, 90, 107]. Changes in microbiota community structure strongly correlate with flock health outcomes [22, 78, 94] with perturbations in certain components (*e.g.* increases *Blautia* spp.) yielding increased performance in broilers and perturbations in others (*e.g.* increases in *Clostridium* spp.) yielding decreased performance [30, 53, 78]. As the microbiome, and constituent microbiota, modulate against animal and human pathogens, produce vitamins, aid in energy acquisition, and aid in host immune system maturation, maintenance of an optimal and uniform microbiome is of utmost importance in sustainable agriculture [61, 103]. The majority of microbiome, microbiota, and metagenomic studies in hens have been performed with broilers. As different dietary compositions, housing protocols, and selective genetic differences between broilers and layers exist, both functional breed groups warrant independent investigation [19].

The overuse of antibiotics for both prophylaxis and as growth promotors was previously common agricultural practice globally and is still commonplace in many countries [16, 33, 102]. This practice has significantly contributed to the silent pandemic of antimicrobial resistance, resulting in the emergence of diverse multidrug resistance plasmids and diminished treatment regimens for clinically relevant pathogens [23, 76, 79]. The European Union banned the use of antibiotics as growth promoters in animal feed in 2006 (Regulation 1831/2003/EC) with many other countries also now prohibiting the addition of antibiotic growth promoters to animal feed [49, 72]. However, the implementation of these bans, has resulted in increased dysbiosis across all affected countries [63]. Whilst the use of antimicrobials in laying hens is much less than in broilers, to avoid antimicrobial residues in eggs there is still a need for strategies which maintain health and improve production [25]. To ensure productivity and maintain animal welfare, mitigation of the emergence and spread of bacterial disease is essential [37]. Current management strategies focus on biosecurity, vaccines, and nutritional supplements [64]. As such, a considerable market gap exists for non-antibiotic microbiota modulators that do not promote clinically relevant antimicrobial resistance and positively aid in growth promotion of food animals such as prebiotics, probiotics, essential oils, and organic acids.

Mannan rich fraction (MRF), derived from the *Saccharomyces cerevisiae* cell wall, is a prebiotic that has been shown to successfully lower pathogen bioburden by binding type-1 fimbriated bacteria (*e.g*., *Escherichia* spp. and *Salmonella* spp.,) via mannose receptors [99], 2015,[62], increase microbiome ecosystem diversity, and improve broiler performance in numerous studies without the use of antibiotic growth promoters [19, 20, 53, 69]. Additionally, parameters such average daily feed intake (ADFI), body weight (BW), egg weight (EW), and egg production (EP) of the hen, are important measures of productivity in the layer industry [6, 7]. A meta-analysis of mannan oligosaccharides supplementation has been reported to improve production rates, feed efficiency, and result in fewer losses from mortality in layers [92]. There is a dearth of information on the microbiota modulating effect of MRF on mature layers. Using prebiotic supplemental approaches instead of indiscriminate antibiotic application allows for both a precision agriculture framework and an antibiotic stewardship framework, where antibiotic intervention is used only if, and when, it is needed.

Microbial taxonomic community studies have offered profound insight into the health status and underlying holobiontic metabolome in animals [87, 88]. Building on previous studies of the hen gastrointestinal microbiota structure, the present study offers a statistically robust insight to the mature layer hen microbiome using, to our knowledge, the largest sample sizes in a prebiotic interventional study with 197 (99 control *vs*. 98 MRF-supplemented) birds. This study also addresses the compositional nature of microbiota [35]. While not utilised in every study, compositions address some biases introduced by relative proportionalities in sequencing data. This approach allows for more robust pairwise comparisons and most accurate multidimensional analyses, such as principal component and coordinate analyses (PCA and PCoA). As the cecum is a rich and diverse ecosystem, the statistical dynamics of rare taxa are often overshadowed by more common species, by accounting for proportionality, the effect of MRF supplementation on rare taxa can be more accurately established. Consequently, the goal of this study was to investigate the effect of MRF supplementation on bacterial diversity, microbiome uniformity, productivity associated taxa, and lay performance.

## Materials and methods

### Animal trial, sample collection and preservation

This trial was performed at a research site in Scotland, United Kingdom and the accommodation and care of animals used in the study was in accordance with Directive 2010/63/EC (https://eur-lex.europa.eu/eli/dir/2010/63/oj) and European Commission Recommendation 2007/526/EC (http://data.europa.eu/eli/reco/2007/526/oj). A total of 344 Shaver female laying hens were randomly allocated to one of two diets (a standard commercial diet or a standard commercial diet supplemented with MRF) and identified by cage. Each treatment was replicated 43 times with four birds per cage using a randomised complete block design. Birds were aged 16 weeks on arrival, the study started with MRF inclusion when the birds were 28 weeks old, and ran for 24 weeks (168 days). The building was supplied with artificial, programmable lights, and forced ventilation. The temperature inside the building was kept between 20 and 25°C as recommended by the breeder. The lighting programme was 16-h light and 8-h dark during each 24-h period throughout the trial. Feed and water were available ad libitum throughout the trial and one feed hopper per cage was provided. General observations of health and temperature recording was carried out twice daily (*am* and *pm*) and feed and water supply was checked at least twice daily. The birds were fed a mash diet throughout the duration of the trial. Experimental diets were calculated to be isonutritive and to meet or exceed the nutrient requirements recommended by the National Research Council (NRC) (1994) for laying hens [21]. The composition and the calculated analyses of the basal diets are presented in Additional file 2: Table S1. Mannan rich fraction (Alltech Biotechnology, Nicholasville, Kentucky) was included in the diet at 800 g/t (0.8 g/kg) until the birds were aged 34 weeks and at 400 g/t (0.4 g/kg) from 34 weeks of age until the end of the laying period.

At day 168 post-MRF introduction (when the birds were aged 52 weeks) the intact cecal pouch of 99 (control) and 98 (MRF-supplemented) randomly selected birds per treatment was excised immediately after humane euthanization. Cecal content was aseptically transferred to tubes containing 20 ml of DNA/RNA shield (Zymo Research, Cambridge Bioscience, UK).

### DNA extraction and sequencing

Cecal content DNA was extracted using a DNeasy Powersoil Pro kit (Qiagen, Germany) according to the manufacturer’s instructions. Genomic DNA concentration, purity and integrity was determined using an Agilent 5400 Fragment Analyzer System (Agilent Technologies, Santa-Clara, CA, USA). Sequencing libraries were generated using NEBNext® Ultra™ DNA Library Prep Kit for Illumina sequencing (NEB, Ipswich, MA, USA). Whole DNA fractions were fragmented by sonication to the size of ~ 350 bp. The DNA fragments were then end-polished, A-tailed, and ligated using a full-length adaptor for Illumina sequencing with further PCR amplification. Each PCR product was purified (AMPure XP system) and library size distributions were established using an Agilent 2100 Bioanalyzer and quantified using real-time PCR. Clustering of the index coded samples was performed on the Illumina cBot Cluster Generation System; then, the library preparations were sequenced on an Illumina HiSeq platform and paired-end reads were generated (Novogene, Cambridge, UK).

### Sequence quality control

Each sequence was quality controlled using Trim-Galore! *v.* 0.6.6 [46] with paired-end default settings and utilising CutAdapt *v.*3.4 [68] and FastQC v.0.11.9 [5]. This procedure yielded 16,210,614 ± 1,801,720 (CI_0.95_ = [15851267, 16569961]; h = 16,165,463) reads per sample.

### Taxonomic classification

Reads associated with small-subunit 16S rRNA were extracted from each sample using PhyloFlash *v*.3.4 [36] to the seventh taxonomic level (-taxlevel 7; species-level) and with a read length of 150 bp. This procedure yielded 162,425 ± 28,565 (CI_0.95_ = [158412, 166439]; η = 159,912) small subunit 16S rRNA genes per sample. As four read alignments per marker gene were required for a positive marker identification, 40,606 ± 7141 (CI_0.95_ = [39603, 41610]; η = 39,978) marker genes were identified per sample.

### Microbiome composition quality control

**Fig. 1:**
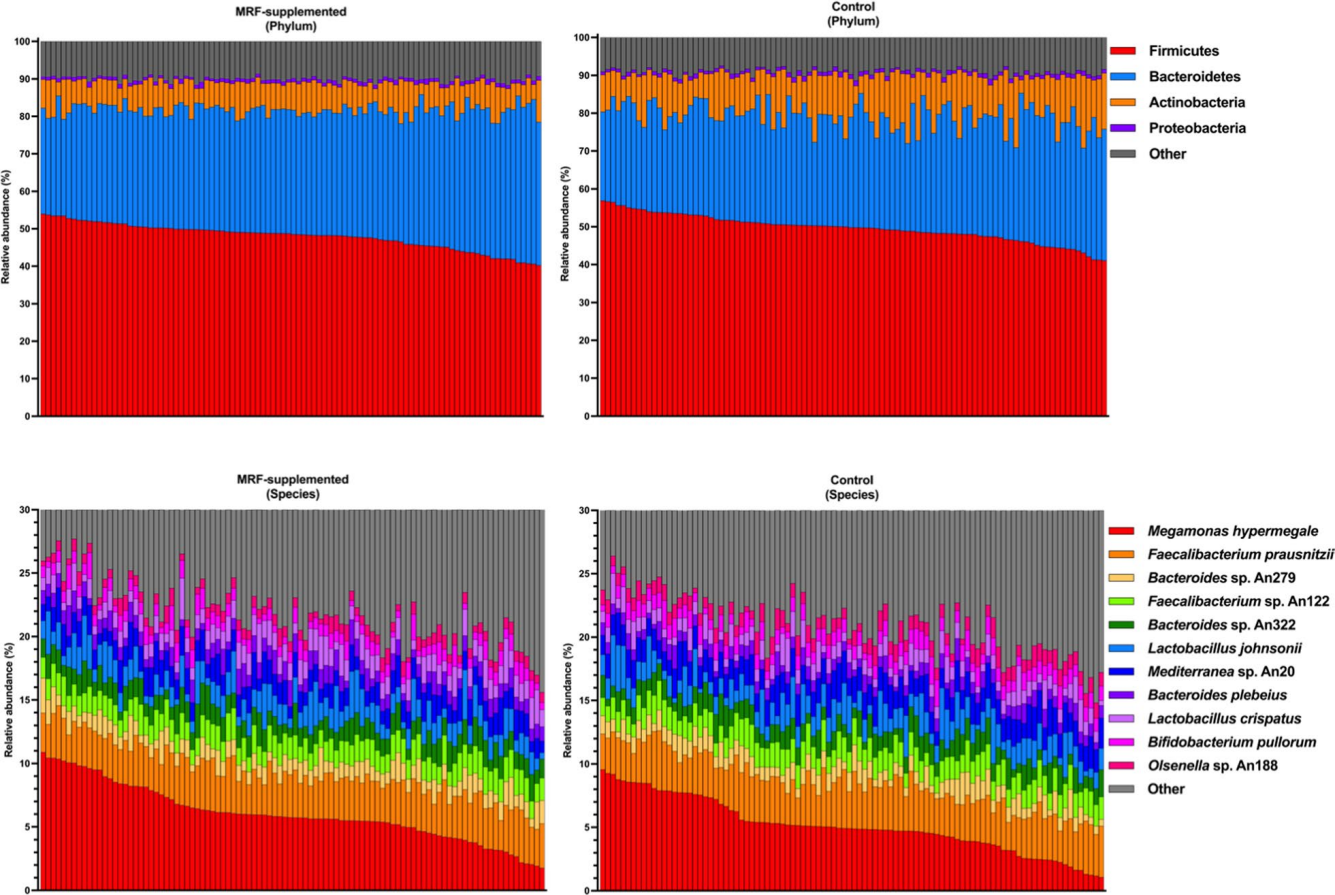
Comparisons of the most abundant taxa in each dietary group

Each of the seven taxonomic datasets (phylum to species) was scaled to 100,000 reads to ensure accurate and comparable analyses (Additional file 2: Tables S2-S7). Each taxon at each rank per experimental group (control and MRF-supplemented) were processed through uniForest *v*.1 [54, 55] with default settings and rescaled to 100,000 reads. The median was found to be the most robust imputer in each process. Data was subjected to closure (scaled to 1) and then multiplied by 100,000 (yielding 100,000 markers per sample) for pairwise comparisons (Additional file 2: Tables S8-S13). Prior to compositional distance analyses (described later in this section), retained data with zero reads were subjected to multiplicative replacement [67] where δ was defined as the count of unique taxa observed across all samples at a given rank. As multiplicative replacement maintains data closure, each sample was then transformed to centre-log ratios. The 20 most abundant species per dietary group are presented in Table 2 (Fig. 1).

### Microbiota pairwise statistics

To determine whether a given taxon was impacted by MRF-treatment, closed data proportions were subjected to Brunner-Munzel test (H_0_:*B* = 0.5;H_A_:*B* ≠ 0.5) powered analyses-of-composition (ANCOM; H_0_:*E*[ln(µ_1_^(1)^)] = *E*[ln(µ_1_^(2)^)];H_A_:*E*[ln(µ_1_^(1)^)] ≠ *E*[ln(µ_1_^(2)^)]) [11, 66]. As ANCOM uses a secondary statistical procedure to determine significance, statSuma *v.*1.3 [54, 55] was used to determine underlying taxonomic distribution features to choose the most appropriate test. To account for both Type I and Type II errors, the critical α was lowered to 0.005 [8] and incidences where *P* ≤ α were considered statistically significant (Additional file 2: Table S14). Taxa with insignificant *P*-values were considered to not have been impacted by treatment.

### Ecological statistics (α-diversity)

**Fig. 2:**
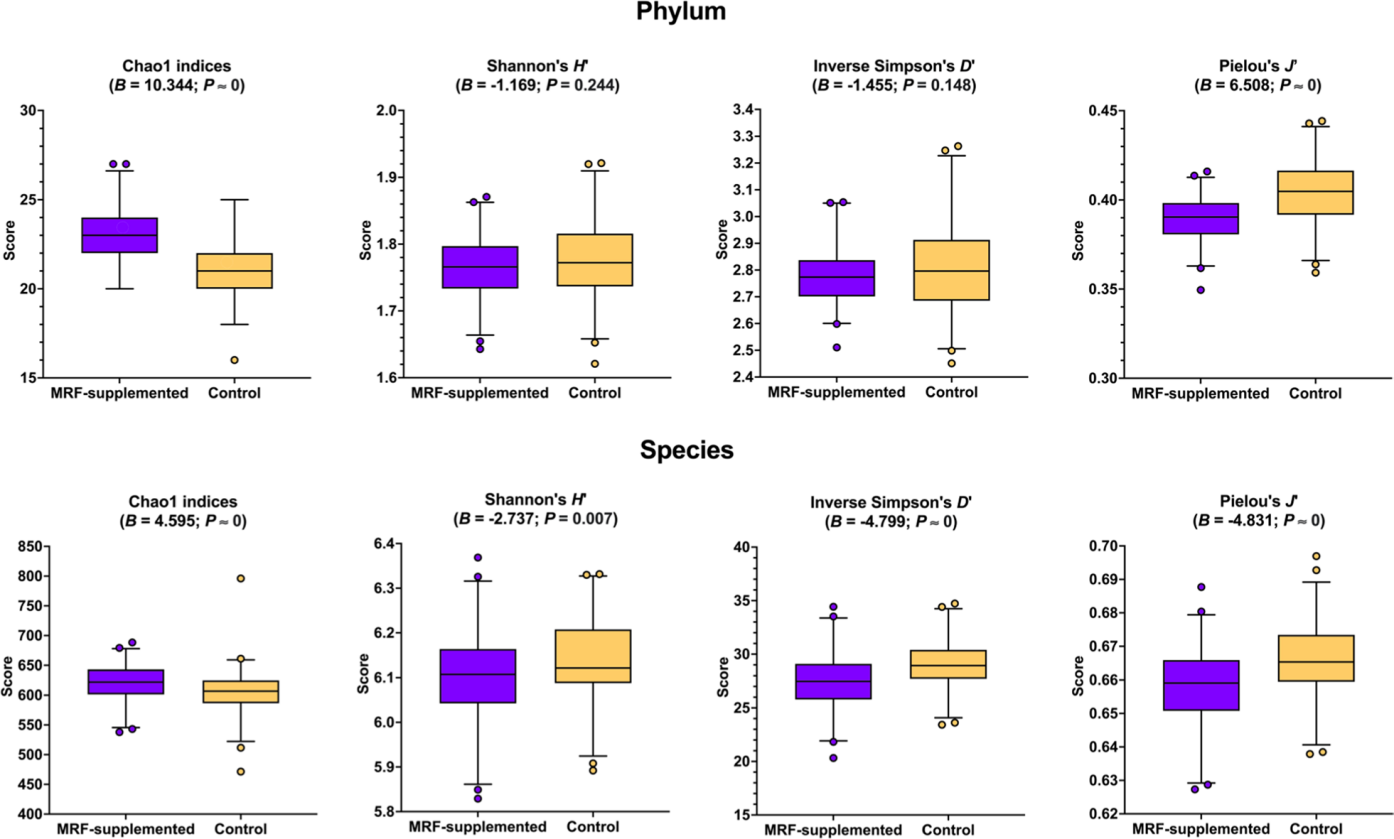
Impact of MRF-supplementation on a-diversity metrics. The horizontal line within each box denotes the mean and tails represent 95% confidence intervals. Observations beyond the 95% CI boundaries are represented as dots. In each plot *B* and *P* refer to the Brunner-Munzel test statistic and its associated *P*-value

For each scaled data sample, four different α-diversity metrics were computed using α-diversity driver functions in the scikit-bio (skbio) library *v.*0.5.8. (http://scikit-bio.org/). Chao1 indices [14] were used to determine taxonomic richness, Shannon’s entropy (*H*’) [96] and reciprocal (inverse) Simpson’s diversity (*D*’^−1^) [97] were used to determine taxonomic diversity, and Pielou’s eveness (*J*’) [83] was used to determine taxonomic evenness (Additional file 2: Table S15). Both diversity measures were used as Shannon’s *H*’ is weighted towards rare taxa and is more sensitive to changes in taxonomic richness, whereas Simpson’s *D*’^−1^ is weighted towards abundant species and is more sensitive to changes in taxonomic evenness [73, 89]. Again, statSuma was used to determine the underlying data Gaussianity and equivariance between treatment groups, where it was determined that the Brunner-Munzel test (H_0_:*B* = 0.5;H_A_:*B* ≠ 0.5) was considered most appropriate. A critical α of 0.005 was selected, and incidences where *P* ≤ α were considered statistically significant (Fig. 2; Additional file 1: Figures S1-S4; Additional file 2: Table S16).

### Ecological statistics (β-diversity)

**Fig. 3:**
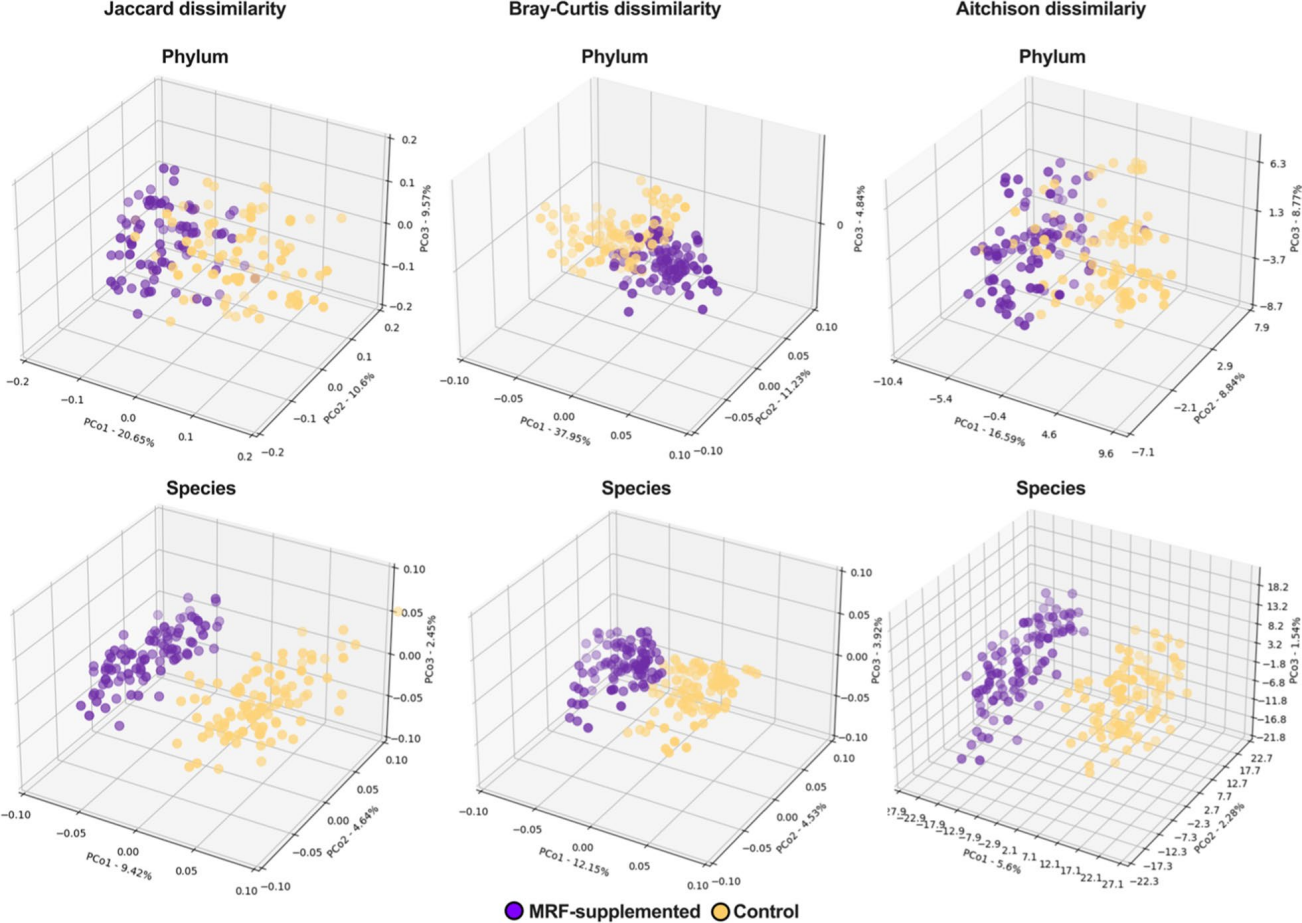
Principal coordinate analyses (b-diversity) Regularly spaced values represented on the *x*, *y*, and *z* axes are distance intervals as defined by their respective dissimilarity indices. The Principal Coordinates (PCo) for each axis are accompanied by their respective explained variances

Jaccard [40], Bray–Curtis [10], and Aitchison [1] dissimilarity matrices were constructed for each taxonomic subset using skbio ordination driver functions using untransformed compositional data matrices. To account for compositionality, data were Hellinger transformed [38] and reclosed prior to the computation of Bray–Curtis dissimilarity matrices. Each dissimilarity matrix was projected using principal coordinate analysis (PCoA,Fig. 3, Additional file 1 Figures S5-S8). Permutational analyses of variance (PERMANOVA; [4]) were used to determine whether control or MRF-supplemented data positions were significantly different via their centroid (*G*) measures between groups (H_0_:*G*_(*a*)_ ~ _(*b*)_,*G*_(*a*)_≁*G*_(*b*)_) with 9,999 iterations. Analyses of similarity (ANOSIM; [15]) were used to determine whether intragroup differences were significantly different to intergroup distances (H_0_:*R* = 0;H_A_:*R* ≠ 0) with 9,999 iterations (Additional file 2: Table S17).

### Ecological statistics (multidimensional analyses of variability)

**Fig. 4:**
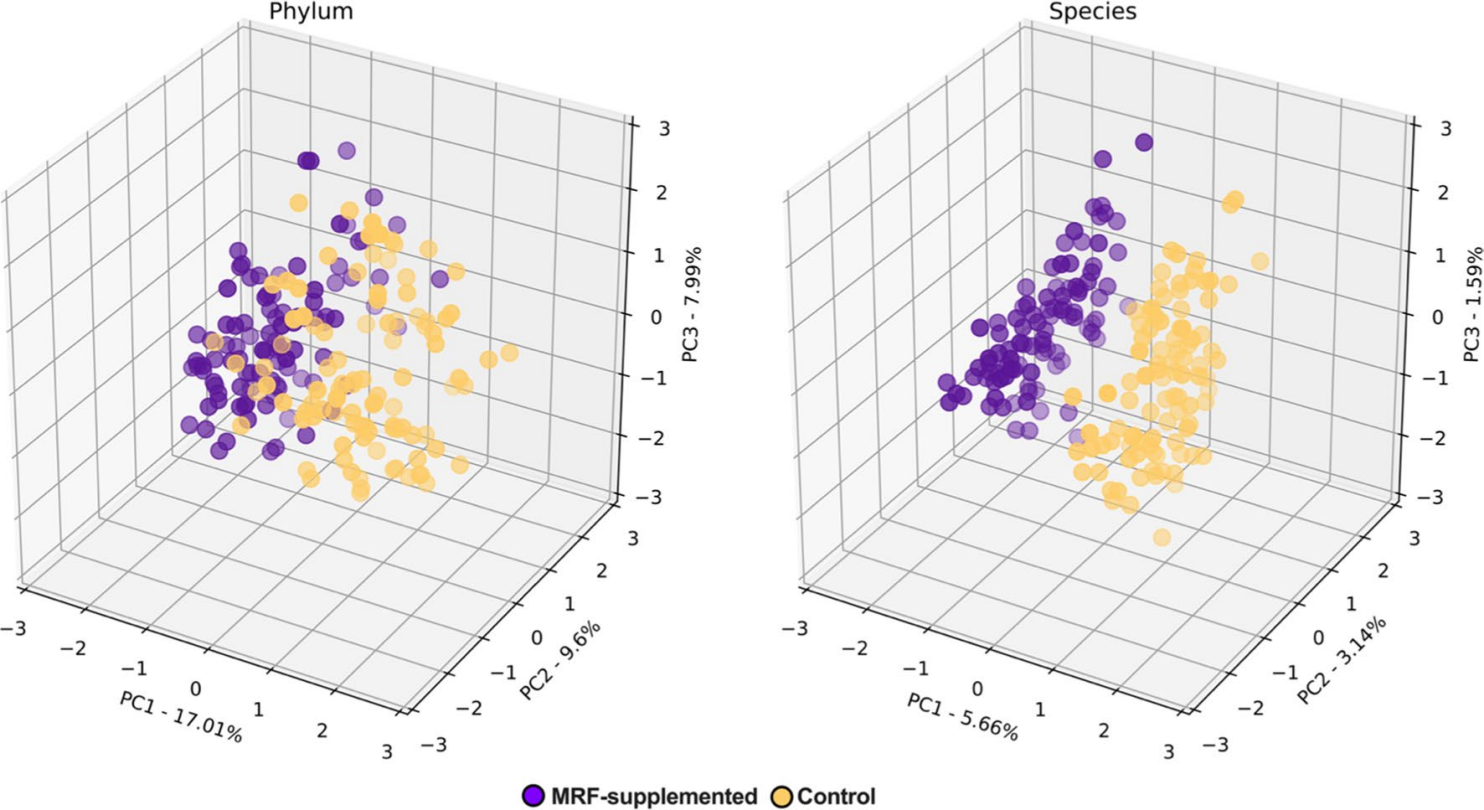
Principal component analyses. Regularly spaced values represented on the *x*, *y*, and *z* axes are standard deviations away from the mean (0) in standardized (*Z*-score) space. The Principal Components (PC) for each axis are accompanied by their respective explained variances

Principal component analyses (PCA; [80]) were used to decompose compositional data (in Aitchison space) and to identify the principal components (PCs) yielding the most variability using the “pca” decomposition driver function from the scikit-learn *v*.0.23.1 Python library [81] (Fig. 4, Additional file 1: Figure S9). Prior to projection, compositional data were whitened to ensure uncorrelated outputs with unit component-wise variances (for more uniform projections between taxonomic ranks) using the “whiten = True” flag offered with the “pca” decomposition driver function.

As above, PERMANOVA were used to determine whether control or MRF-supplemented data positions were significantly different (H_0_:*G*_(*a*)_ ~ *G*_(*b*)_;H_A_:*G*_(*a*)_≁*G*_(*b*)_) with 9,999 iterations and ANOSIM were used to determine whether intragroup differences were significantly different to intergroup distances (H_0_:*R* = 0;H_A_:*R* ≠ 0) with 9,999 iterations. Again, a critical α of 0.005 was selected and instances where *P* ≤ α were considered statistically significant (Additional file 2: Table S18).

#### Assessment of uniformity using *n*-dimensional distances

Sample uniformity was assessed using density-based spatial clustering of applications with noise (DBSCAN) [29] utilizing the “dbscan” clustering driver function from the scikit-learn *v*.0.23.1 Python library. The DBSCAN algorithm was performed using default functions except for the ε value, which was individually derived from the geometric mean of all nonzero Aitchison pairwise distances for the MRF-supplemented and control datasets for each respective taxonomic rank. The geometric mean was chosen over the arithmetic mean to limit attraction from extreme values and was chosen over the median to facilitate a more punitive experiment by allowing ε to move from the exact centre. Contingency tables were constructed by counting the number of inliers *vs.* outliers for MRF-supplemented and control samples for each taxonomic rank. Each contingency table was subjected to a two-tailed Fisher’s exact test (H_0_:p̂_*a*_ = p̂_*b*_:H_A_:p̂_*a*_* ≠ *p̂_*b*_) [31] where a *P* ≤ 0.005 was considered statistically significant and direction was determined by comparing proportions (*P* ≤ 0.005; p̂_*a*_ > p̂_*b*_: outliers are significantly increased in treatment group *a* and *vice-versa*) (Additional file 2: Table S19).

#### Assessment on quality and productivity

**Fig. 5:**
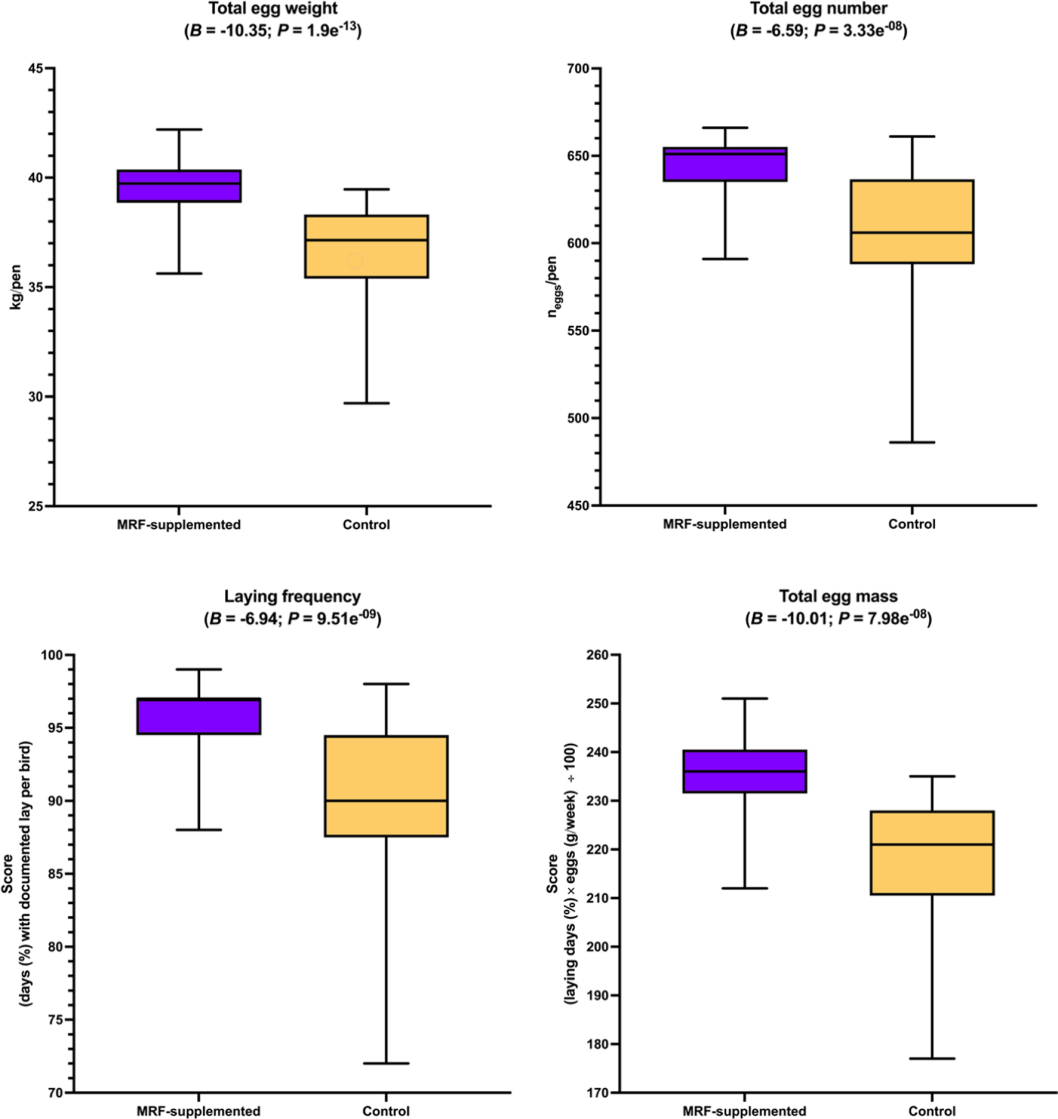
Impact of MRF-supplementation on productivity factors. The horizontal line within each box denotes the mean and tails represent 95% confidence intervals. No observations exceeded the 95% CI intervals. In each plot *B* and *P* refer to the Brunner-Munzel test statistic and its associated *P*-value

**Fig. 6:**
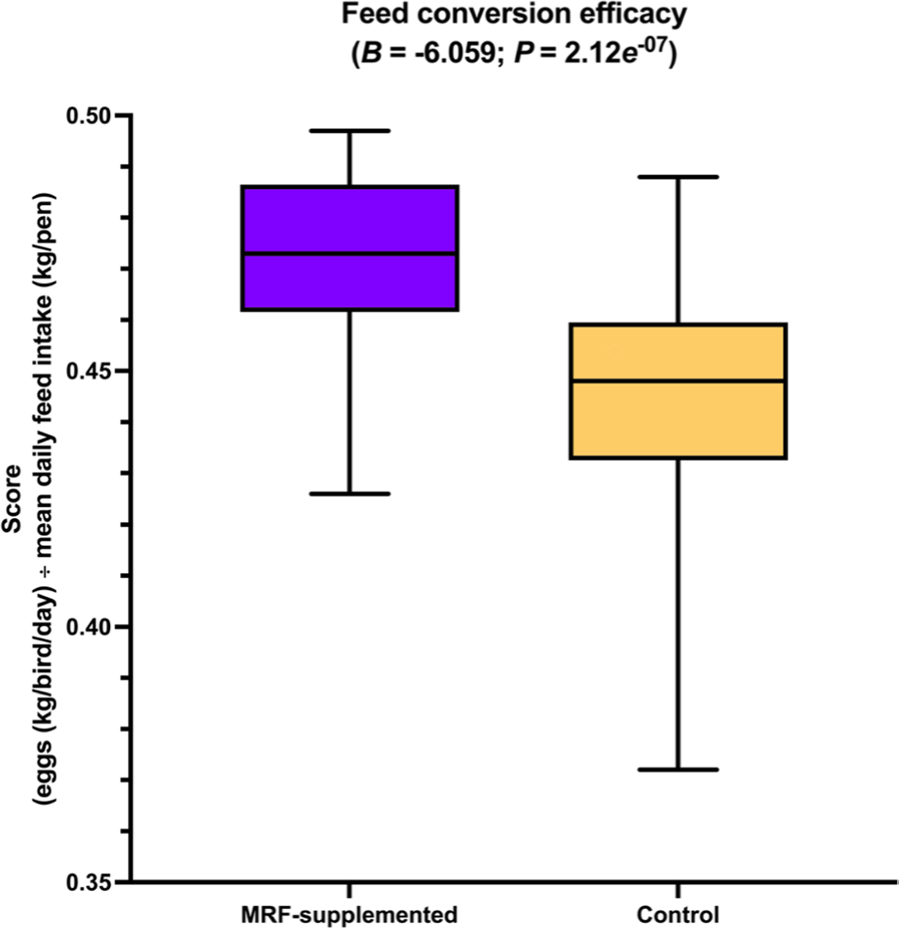
Impact of MRF-supplementation on feed conversion efficiency. The horizontal line within each box denotes the mean and tails represent 95% confidence intervals. No observations exceeded the 95% CI intervals. In each plot *B* and *P* refer to the Brunner-Munzel test statistic and its associated *P*-value

Measures of egg quality (shell strength (kg/m^3^), shell thickness (mm), yolk colour score (unitless) were taken for one egg per pen at 7 timepoints (days 0, 30, 58, 86, 114, 142, and 168). Average egg weight (g)), hen productivity (total egg quantity (*n*), total egg weight (kg), total egg mass (g/bird/day), laying frequency (percentage of birds with a successful lay per day (%)), feed economics (average food intake (AFI (kg/bird/day)) and feed conversion efficiency (FCE (score))) were assessed for each pen for every four week period (0–4, 4–8, 8–12, 12–16, 16–20, 20–24) and aggregated from week 0 to week 24, where each pen contained between three and four birds (Additional file 2: Table S20). For pens containing three birds (pens 2 and 61; both were MRF-supplemented), total egg mass, total egg quantity, total egg weight, combined bird weight at week 0, and combined bird weight at week 24 were standardised by multiplying by 1.333. Multiplication by this factor equivocates to dividing by 3 then multiplying by 4. One MRF-supplemented pen (pen 147) was reported to have an unusually high albumin height (11.5 mm) and corresponding Haugh unit (104.5). As these units were approximately twice the median of the remainder of their respective distributions (η_albumin height_ = 4.95 mm; η_Haugh unit_ = 67.25), the reported values were divided by two to simulate standard observations. The combined weight of each bird in a pen (kg) at the beginning of the trial and end of the trial was also collected to ensure accurate comparability. By adjusting this data, we were able to maintain an equal number for each correlation analysis. This allowed us to better compare outcomes (using ranked correlation analyses, discussed below) without introducing statistical bias. To enumerate the number of eggs laid per day, the total number of eggs were divided by the duration of the trial (168 days) then divided by the laying frequency (as a proportion), and finally dividing by 4 to represent the number of birds per pen. In all instances, this number was 1, suggesting that instances of multiple eggs being laid in a single day by any bird were highly unusual (if such events occurred at all). A total of 25 pens were used for the MRF-supplemented treatment and 25 for the control treatment. Measures for MRF-supplemented birds were compared to control diet birds using Brunner-Munzel tests (H_0_:*B* = 0.5;H_A_:*B* ≠ 0.5). Again, a critical α of 0.005 was selected and instances where *P* ≤ α were considered statistically significant (Table 3; Fig. 5). For the purposes of measuring productivity and quality feature variability, a simple ratio was devised to determine how dispersed a given dataset is, hereafter referred to as the "relative dispersion factor” and represented by the archaic division symbol ϙ (“qoppa” (/ˈkopa/ [ˈko.pa])), where ϙ is simply the ratio between dispersion indices from univariate data series. The relative dispersion factor was calculated for each feature and were assessed to determine whether MRF-supplemented factors were more variable (ϙ > 1) or less variable (ϙ < 1) than control factors. As ϙ is a multiplicative measure with reference to the control dataset, an instance where, for example, ϙ = 0.5 means the MRF-supplemented dataset is half as dispersed as the control data and instances where ϙ = 2 would mean that the MRF-supplemented data is twice as dispersed as the control data. Finally, to determine ϙ statistical significance, a Levene’s test (H_0_:σ^2^_(*a*)_ = σ^2^_(*b*)_;H_A_:σ^2^_(*a*)_ ≠ σ^2^_(*b*)_) [57] was calculated between MRF-supplemented and control features (Table 3,Fig. 6). Instances where *P* ≤ 0.05 were considered statistically significant. A critical α of 0.05 is used instead of 0.005 to reflect how unequal variances are commonly assessed, where a lower critical α is likely to yield false negatives [8]

#### Associations between taxa and quality and productivity factors

The centre log ratio for each taxon from each pooled composition was computed and assessed for association with quality and productivity factors using a Spearman’s *ρ* (H_0_:*X*_(*m)*_ ∝ *x*_(*n*)_);H_0_:*X*_(*m)*_ ∝ /*x*_(*n*)_)). A *ρ* ≥ 0.3 was considered to be positively correlated, a *ρ* ≤ -0.3 was considered negatively correlated, and instances where -0.3 < *ρ* > 0.3 were not considered to be associated. A critical α of 0.005 was selected and instances where *P* ≤ α were considered statistically significant. However, as correlations using compositional data skew negatively [35], only significant correlations that were also significantly impacted (as confirmed by ANCOM) were considered for interpretation (Additional file 2: Table S21).

#### Phylogeny construction

**Fig. 7:**
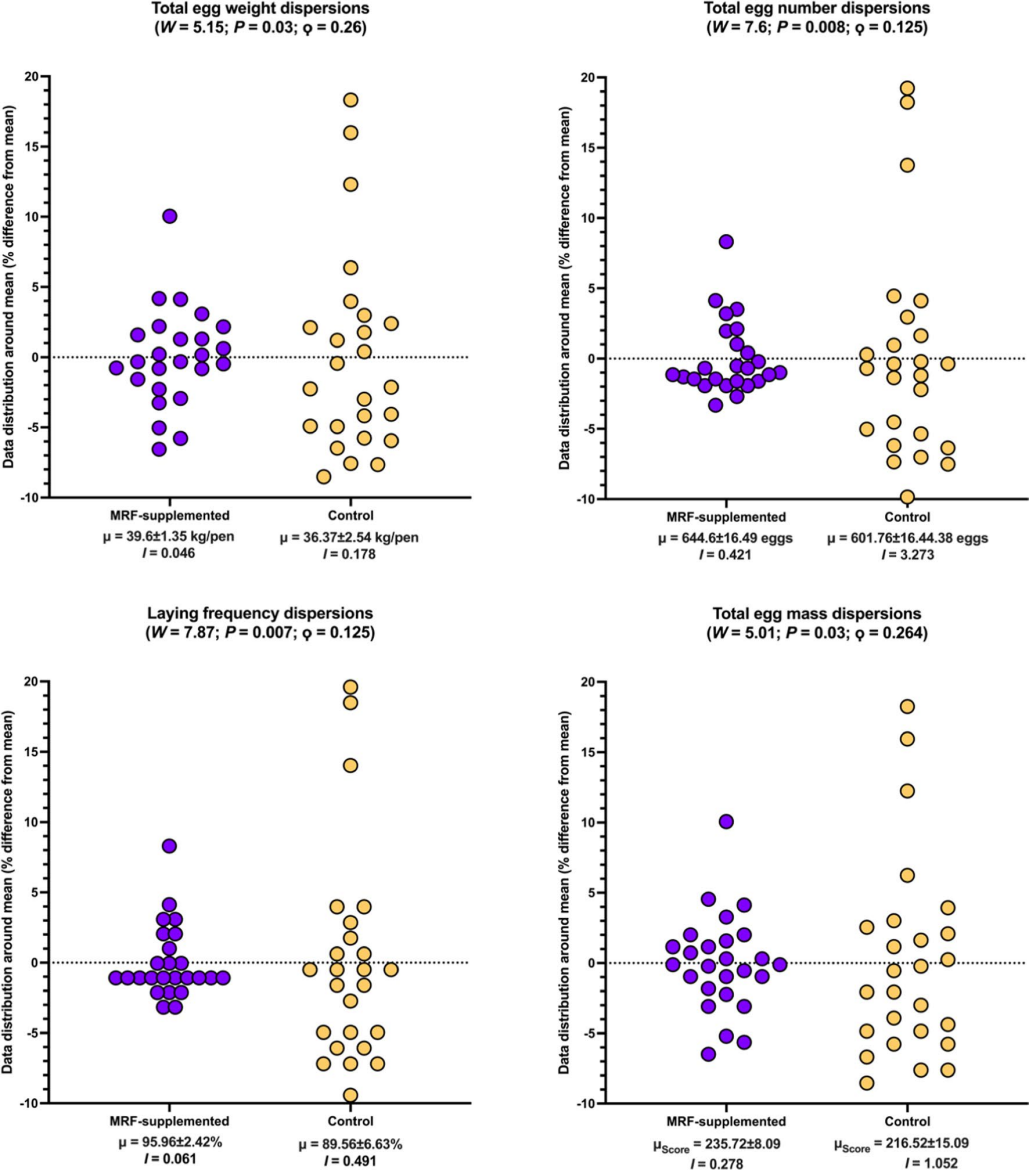
Impact of MRF-supplementation on production consistency. The dashed line at 0 represents the mean of each productivity factor. Data from the MRF-supplemented pens were statistically closer to their respective means with significantly less variance

An accurate phylogeny was required to best interpret results (Fig. 7). Representative genomes for each species level taxa (except *incertae sedis* species) were downloaded from NCBI Assembly [43]. Each genome was annotated using Prokka *v*1.14.6 [93]. Each sequence in each annotated genome (in amino acid format) was searched against each other genome using BLASTP *v.*2.13.0 with an *e*-value stringency score of *E* ≤ 1*e*^−20^ in output format 6 using the “qseqid sseqid evalue pident qstart qend qlen sstart send slen” flags. The resultant BLAST output table was annotated with query and subject coverage scores using the formulae:

$${\text{cov}}_{\text{q}}\text{=} \left(\frac{\text{qend - qstart - 1}}{\text{qlen}}\right)\text{;} {\text{cov}}_{\text{s}}\text{=} \left(\frac{\text{send - sstart }\text{- 1}}{\text{slen}}\right)$$

where: cov_*q*_:Query sequence coverage, cov_*s*_:Subject sequence coverage, qend:End of query sequence alignment (amino acid position), send:End of subject sequence alignment (amino acid position), qstart:Start of query sequence alignment (amino acid position), sstart:Start of subject sequence alignment (amino acid position), qlen:Length of query sequence (amino acids), slen:Length of subject query sequence (amino acids).

One amino acid was subtracted from the numerator formulae to account for the minimum alignment position of 1. Instances where percentage identity (pident) ≥ 30%, cov_*q*_ ≥ 0.8, and cov_*s*_ ≥ 0.8 were considered *bona fide* orthologs and extracted as an edge list. The resultant edge list was clustered using MCL *v.*14.137 [24] using the “–abc" flag and a default inflation value of 2. Each cluster that contained only single copy orthologs (where *n*_taxa_ = *n*_sequences_) were used to develop a phylogenetic signal. Sequences from each cluster were extracted from their respective genomes using “blastdbcmd” (from the BLAST suite) and each cluster was subjected to multisequence alignment using Muscle *v.*3.8.1551 [27] with default parameters. Each alignment was quality trimmed with TrimAL *v*.1.4.rev15 [12] using the “-automated1” flag and concatenated into a superalignment using FASconCAT *v.*1.05.1 [47]. The superalignemt was subjected to 10,000 bootstrap replicates using IQ-TREE *v*.2 [74] where the LG model of protein evolution [51] was determined to be most accurate. The resultant newick file was visualised and further annotated using iToL *v*.5 [56]. The root of the phylogeny was set at the branch representing the most recent common ancestor to Actinobacteria and Firmicutes. This branch was chosen to reflect the hypothesis of Terrabacteria being the earliest diverging extant bacterial superphylum [17]. In our dataset, one taxon (*Euhalotece* sp. KZN 001; Cyanobacteria) should also be included in this clade, however it was placed within the Gracilicutes superphylum. The Synergistota *Cloacibacilus* sp. An23 was correctly placed as an outgroup of Terrabacteria [17, 42]. Rooting at Synergistota, however, resulted in the grouping of Firmicutes with Gracillicutes and not as a sister group of Actinomycetes so this was not utilised. Deferribacterota and Campylobacterota are correctly placed as sister taxa as early diverging clades from the Proteobacteria [77, 105]. Spirochaeta were also correctly placed closer to the FCB (Bacteroides) clade than Proteobacteria. All taxa where correctly placed to their correct Phyla. Within Bacteroides, all taxa from the same genus are placed as sister taxa to each other suggesting correct phylogenetic placement. In Actinobacteria, *Colinsella* sp. AF14-35 is placed as the outgroup of *Colinsella* sp. An7 and *Enorma massiliensis*. However, *E. massiliensis* was previously classified as *Colinsella* which may indicate a very recent evolutionary divergence which is not fully captured using our phylogeny. Firmicutes was correctly split into two distinct groups, with Erysipelotrichia and Bacilliales forming one group, itself placed as a sister taxon to the Clostridia-Negativicutes group. The members of Lachnospiraceae (Clostridia) are within the correct family, however genera partition was observed. This may be due to some taxa from *Ruminococcus* being incorrectly assigned to this genus with *Blautia* being a closer relative [59, 60]. The class Negativicutes (Firmicutes) were reportedly clustered together, however instead of being placed as sister taxa to all Clostridia, two Clostridia taxa (*Desulfitobacterium hafniense* and *D. dehalogenans*) were reported as diverging earlier than Negativicutes, however this may be an artefact due to the comparatively low GC content this genus has compared to other Clostridia [26, 50, 104]. The *incertae sedis* taxon “bacterium 1xD42-67” (GCA_003612335.1) was placed as a sister taxon to *Clostridium phoceensus*, matching its closest matching type species (average nucleotide identity = 83.61%) on NCBI Assembly. This phylogeny was used to display all relevant statistical test results pertaining to MRF-supplementation.

## Results

### Effect of mannan-rich fraction on microbial populations

The phyla Firmicutes, Bacteroidetes, Actinobacteria, and Proteobacteria accounted for the majority of both control and MRF-supplemented datasets, accounting for 98.89% and 98.83%, respectively (Additional file 2: Table S8; Fig. 1). Following MRF-supplementation, 19 of the 35 identified Phyla were significantly different in abundance compared to the control (Additional file 2: Table S14). Of these, six definitive phyla (Bacteroidetes, Chloroflexi, Cyanobacteria, Spirochaetes, Synergistetes, Tenericutes) and the *incertae sedis* taxa were significantly higher in abundance in the MRF-supplemented dataset, while nine definitive phyla (Actinobacteria, Aquificae, Chrysiogenetes, Coprothermobacterota, Deinococcus-Thermus, Firmicutes, Gemmatimonadetes, Ignavibacteriae and Thermotogae) and three *candidatus* phyla (*Ca*. Melainabacteria, *Ca.* Nomurabacteria, and *Ca.* Saccharibacteria) were significantly lower compared to the control group. Interestingly, three of the four major constituent phyla were affected by MRF supplementation, whereby Actinobacteria and Firmicutes were significantly lower and Bacteroidetes were significantly higher with MRF supplementation (Fig. 1).

Of the 20 most abundant species in the MRF-supplemented and control samples, 18 were shared (*Bacteroides coprocola, Bacteroides plebeius, Bacteroides salanitronis, Bacteroides* sp. An279, *Bacteroides* sp. An322, *Bifidobacterium pullorum, Cloacibacillus* sp. An23, *Faecalibacterium prausnitzii, Faecalibacterium* sp. An122, *Lactobacillus crispatus, Lactobacillus johnsonii, Lactobacillus reuteri, Lactobacillus salivarius, Mediterranea* sp. An20, *Megamonas hypermegale, Olsenella* sp. An188, *Olsenella* sp. An293, *Prevotella* sp. G:487 50 53), with two species unique to the 20 most abundant taxa in the MRF-supplemented dataset (*Megamonas funiformis* and *Lactobacillus amylovorus*) and two species unique to the control sample 20 most abundant taxa (*Blautia* sp. OM05-6 and *Collinsella* sp. An268) (Table 1). Within the top 20, only two species were significantly impacted by MRF treatment, *Cloacibacillus* sp. An23 and *Prevotella* sp. AG:487 50 53 (both were higher in MRF-supplemented samples; *P* < 0.005), suggesting that the most abundant taxa are largely unaffected by MRF-supplementation, with most of the impact observed in rarer species.

**Table 1:** The 20 most abundant species per treatment group

	MRF-supplemented birds	Control birds		
Rank	Species	η_%_	Species	η_%_
1	*Megamonas hypermegale*	5.754	*Megamonas hypermegale*	4.916
2	*Faecalibacterium prausnitzii*	3.334	*Faecalibacterium prausnitzii*	3.377
3	*Faecalibacterium* sp. An122	1.724	*Mediterranea* sp. An20	1.888
4	*Mediterranea* sp. An20	1.652	*Faecalibacterium* sp. An122	1.781
5	*Lactobacillus johnsonii*	1.652	*Lactobacillus johnsonii*	1.640
6	*Bacteroides* sp. An322	1.459	*Bacteroides* sp. An322	1.369
7	*Lactobacillus crispatus*	1.382	*Bacteroides* sp. An279	1.269
8	*Bacteroides* sp. An279	1.203	*Bifidobacterium pullorum*	1.074
9	*Bacteroides plebeius*	1.102	*Lactobacillus crispatus*	1.065
10	*Bifidobacterium pullorum*	0.928	*Olsenella* sp. An188	1.055
11	*Olsenella* sp. An188	0.781	*Lactobacillus reuteri*	0.900
12	*Lactobacillus reuteri*	0.685	*Bacteroides plebeius*	0.883
13	***Cloacibacillus* sp. An23**	0.587	*Olsenella* sp. An293	0.612
14	*Megamonas funiformis*	0.552	*Lactobacillus salivarius*	0.597
15	***Prevotella* sp. AG:487 50 53**	0.543	*Blautia* sp. OM05-6	0.503
16	*Bacteroides coprocola*	0.531	*Bacteroides salanitronis*	0.467
17	*Olsenella* sp. An293	0.501	*Bacteroides coprocola*	0.452
18	*Bacteroides salanitronis*	0.460	***Prevotella* sp. AG:487 50 53**	0.375
19	*Lactobacillus salivarius*	0.431	***Cloacibacillus* sp. An23**	0.344
20	*Lactobacillus amylovorus*	0.412	*Collinsella* sp. An268	0.338

Species are shown alongside their median relative abundance per treatment group. The two species that were significantly affected (*Cloacibacillus* sp. An13 and *Prevotella sp.* AG:487 50 53

In total, 46 species level taxonomic assignments were significantly more abundant in MRF supplemented birds (Additional file 2: Table S14): one within Actinobacteria (*Ilumatobacter fluminis*), 20 within Bacteroidetes, one within Deferribacteres (*Mucispirillum schaedleri*), one within Elusimicrobia (*Elusimicrobium* sp. An273), 17 within Firmicutes, three within Proteobacteria (one Betaproteobacteria (*Sutterella* sp. AM11-39) and two Gammaproteobacteria (*Succinatimonas hippei* and *Methylohalobius crimeensis*)), two within Spirochaetes (*Sphaerochaeta coccoides* and *Treponema* sp. UBA6367), and one within Synergistetes (*Cloacibacillus* sp. An23). Within Bacteroidetes, ten species were significantly more abundant in the MRF-supplemented dataset within the genus *Prevotella* (*P. albensis, P. colorans, P. oralis, P. pectinovora, P. pleuritidis, P. scopos, Prevotella* sp. 885, *Prevotella* sp. AG:487 50 53, *Prevotella* sp. oral taxon 299, *Prevotella* sp. oral taxon 820), four within *Bacteroides* (*B. acidifaciens, B. dorei, B. fluxus,* and *B. ovatus*), and all other significantly more abundant Bacteroidetes species were monotypic per genus (*Alistipes shahii*, *Barnesiella* sp. WM24, *Dysgonomonas* sp. BGC7, *Parabacteroides* sp. An277, *Parapedobacter indicus*, and *Pontibacter actiniarum*). Within Firmicutes, four significantly more abundant species were observed within the MRF-supplemented samples and were assigned to the Clostridiaceae (*Butyricicoccus porcorum, Clostridium phoceensis, Clostridium* sp. AF36-4, and *Clostridium* sp. OF03-18AA), seven were within Lachnospiraceae (*Anaerobutyricum hallii, Anaerostipes hadrus, Anaerostipes* sp. 494a, *Anaerotignum neopropionicum, Coprococcus catus, Dorea* sp. OM02-2LB, *Tyzzerella* sp. An114), and three were within the Ruminococcaceae (*Flavonifractor plautii, Flavonifractor* sp. An92, and *Ruminococcus* sp. AF18-22). The remaining Firmicutes species which were more abundant were monotypic for their respective families *Staphylococcus saprophyticus* (Staphylococcaceae), *Megamonas funiformis* (Selenomonadaceae), and *Megasphaera hexanoica* (Veillonellaceae). Comparatively, nine Actinobacteria (*Aeriscardovia aeriphila, Bifidobacterium magnum, Bifidobacterium pseudocatenulatum, Bifidobacterium pseudolongum, Bifidobacterium scaligerum, Collinsella* sp. AF14-35, *Collinsella* sp. An7, *Enorma massiliensis*, and *Paraeggerthella hongkongensis*), one Bacteroidetes (*Alistipes putredinis*), 27 Firmicutes, and one Proteobacteria (*Campylobacter jejuni*) were significantly lower in the MRF-supplemented samples (Additional file 2: Table S16). Within Firmicutes, two within Acidaminococcaceae (*Phascolarctobacterium succinatutens* and *Succinispira mobilis*), one within Clostridiaceae (*Butyricicoccus* sp. N15.MGS-46), one within Erysipelotrichaceae (*Faecalitalea cylindroides*), one within Eubacteriaceae (*Anaerofustis stercorihominis*), nine within Lachnospiraceae (*Blautia hominis*, *Blautia* sp. AF19-13LB, *Blautia* sp. KGMB01111, *Blautia* sp. N6H1-15*, Lachnotalea glycerini, Roseburia faecis, Roseburia* sp. AM16-25, *Roseburia* sp. UNK.MGS-15, and *Tyzzerella nexilis*), one within Lactobacillaceae (*Lactobacillus equigenerosi*), two within Peptococcaceae (*Desulfitobacterium dehalogenans* and *Desulfitobacterium hafniense*), five within Peptostreptococcaceae (*Clostridioides mangenotii, Intestinibacter bartlettii, Paeniclostridium sordellii, Romboutsia maritimum*, and [Clostridium] *dakarense*), four within Ruminococcaceae (*Anaerotruncus* sp. AF02-27, *Ruminococcus* sp. AF14-5, *Ruminococcus* sp. OM05-7, and *Ruminococcus* sp. Zagget7), and one within Sporomusaceae (*Sporomusa sphaeroides*) were significantly lower in MRF supplemented birds. It should be stated that several significantly impacted taxa (specifically those denoted using “An” (*e.g*., *Flavonifractor* sp. An92) were first properly described in a single large scale, high quality culturomic and metagenomic study of hen caecal anaerobes [70]

### Effect of mannan-rich fraction on α-diversity

With regards to taxon richness, Chao1 indices were significantly higher and Pielou’s *J’* were significantly lower in MRF-supplemented birds (Fig. 2; Additional file 1: Figs. 1–4; Additional file 2: Table S16). Regarding diversity, no significant difference was observed at the phylum rank whereas Simpson’s *D*’^−1^, but not Shannon’s *H*', was significantly lower at the species rank in MRF-treated birds.

### Effect of mannan-rich fraction on β-diversity

Regardless of ordination matrix (Aitchison distance, Bray–Curtis dissimilarity, or Jaccard indices), considerable separation was visually observed at all taxonomic ranks using PCoA (Fig. 3; Additional file 1: Figures S5-S7), with explained variances of 12.58%-18.91% (16.01 ± 2.68%) for principal coordinate (PCo) 1, 4.37%-8.53% (5.89 ± 1.6%) for PCo2, and 3.02%-7.3% (4.95 ± 1.79%) for PCo3. When taken together, the sum of PCos per taxonomic rank accounted for 20.66%-32.98% (26.85 ± 5.39%) of total explained variance. The separability of PCoA results were statistically confirmed with PERMANOVA and ANOSIM where even greater divergence was observed (0.493 ≤ *R* ≤ 0.694) (Additional file 2: Table S17). Considerable variances were also observed when assessed using PCA (Fig. 4; Additional file 1: Figure S8) with explained variances ranging between of 5.66%-17.01% (11.38 ± 4.61%) for principal component 1 (PC1), 3.14%-9.6% (5.22 ± 2.52%) for PC2, and 1.59%-7.99% (4.18 ± 2.42) for PC3. Taken together, the sum of PCs per taxonomic rank accounted for 10.39–34.6% (20.78 ± 9.34%) of total explained variance. These findings were statistically verified by significant PERMANOVA and ANOSIM results, suggesting considerable centroid separation (Additional file 2: Table S18). All ANOSIM *R* scores were moderately to highly divergent (0.35 ≤ *R* ≤ 0.549) further confirming greater separation between groups and highlighting lower intragroup variation when compared with intergroup variation.

### Effect of mannan-rich fraction on uniformity

Significantly greater uniformity was observed for the species taxonomic rank with 21 fewer outliers observed (10 *vs.* 31; 10.2% *vs.* 31.3%) in MRF-supplemented group compared with the control (Additional file 2: Table S19). As the species level is intrinsically the most variable, these results demonstrate a fundamental difference in data composition.

### Effect of mannan-rich fraction on egg quality and layer productivity

The addition of MRF resulted in no statistically significant differences in egg quality (shell strength, shell thickness, yolk colour score, or average egg weight) or bird weights. However, MRF-addition was observed to significantly result in improved productivity factors (total egg weight, total egg numbers, total egg mass, and laying frequency) over the 24 weeks of this trial, representing 6.79%-7.78% greater productivity (Table 2; Fig. 5). Feed conversion ratio was also significantly better (+ 5.58%) following MRF supplementation (Table 2; Fig. 6). Decreases in statistical dispersion were also observed in the four productivity factors (0.125 ≤ ϙ ≤ 0.264) representing an approximate 4-to-eightfold lower variability following MRF supplementation (Table 3; Fig. 7).

**Table 2:** Impact of MRF-supplementation on production, quality, feed utility

	Factor	MRF-supplemented			Control			Statistical observations		
		**μ**	**σ**	**η**	**μ**	**σ**	**η**	***B***	***P*-value**	**Observed effect**
Bird weight	Week 0 (kg/pen)	7.278	0.32	7.21	7.30	0.33	7.26	0.28	0.783	Lower with MRF
	Week 24 (kg/pen)	8.010	0.39	8.01	8.00	0.40	8.03	0.01	0.993	Lower with MRF
	D_weight_ (kg/pen)	0.723	0.28	0.69	0.70	0.26	0.76	−0.06	0.955	Lower with MRF
Feed utility	**Feed conversion efficiency**	**0.472**	**0.02**	**0.47**	**0.44**	**0.03**	**0.45**	**−6.06**	**2.12*e***^**−07**^	**Higher with MRF**
	Mean feed intake (g/bird/day)	0.125	0.003	0.13	0.122	0.003	0.12	−2.02	0.049	Higher with MRF
Productivity	**Proportional laying days (%)**	**95.960**	**2.42**	**97.00**	**89.56**	**6.63**	**90.00**	**−6.94**	**9.51*e***^**−09**^	**Higher with MRF**
	**Total egg count (*n*)**	**644.600**	**16.49**	**651.00**	**601.76**	**44.38**	**606.00**	**−6.59**	**3.33*e***^**−08**^	**Higher with MRF**
	**Total egg mass (Score)**	**235.720**	**8.10**	**236.00**	**216.52**	**15.09**	**221.00**	**−10.0**	**7.98*e***^**−13**^	**Higher with MRF**
	**Total egg weight (kg/pen)**	**39.601**	**13.53**	**39.728**	**36.37**	**2.545**	**37.146**	**-10.3**	**1.90*e***^**−13**^	**Higher with MRF**
Quality	Albumin height (mm)	4.86	0.79	5.00	4.83	0.92	4.90	−0.18	0.857	Higher with MRF
	Haugh unit (unitless)	64.93	8.19	66.90	65.25	9.59	66.40	0.18	0.857	Higher with MRF
	Mean egg weight (g)	62.60	4.38	64.60	62.00	4.91	62.70	−0.52	0.603	Higher with MRF
	Shell strength (kg/m^3^)	3.54	0.93	3.69	3.88	0.69	3.92	1.38	0.174	Lower with MRF
	Shell thickness (mm)	0.35	0.04	0.36	0.32	0.04	0.31	−2.57	0.013	Higher with MRF
	Yolk colour (unitless)	2.88	0.52	3.00	3.12	0.59	3.00	1.44	0.157	No difference

Significant results (*P* < 0.005) are emboldened. Regarding statistical nomenclature, μ, σ, η, and *B*, refer to mean, standard deviation, median, the and Brunner-Munzel test statistic, respectively

**Table 3:** Impact of MRF-supplementation on productivity, quality, and feed utility consistency

	Factor	MRF-supplemented			Control			Statistical observations			
		μ	σ^**2**^	***I***	μ	σ^**2**^	***I***	**ϙ**	***W***	***P*-value**	**Observed effect**
Bird weight	Week 0 (kg/pen)	7.278	0.100	0.01	7.304	0.109	0.015	0.915	0.053	0.819	Lower with MRF
	Week 24 (kg/pen)	8.010	0.155	0.02	8.003	0.161	0.020	0.968	0.012	0.912	Lower with MRF
	D_weight_ (kg/pen)	0.723	0.081	0.11	0.699	0.070	0.009	1.123	0.095	0.759	Higher with MRF
Feed utility	Feed conversion efficiency	0.472	3.11*e*^−04^	6.59*e*^−04^	0.442	7.58*e*^−04^	0.002	0.384	1.145	0.290	Lower with MRF
	Mean feed intake (g/bird/day)	0.125	1.22*e*^−05^	9.76*e*^−05^	0.122	1.00*e*^−05^	8.16*e*^−05^	1.196	0.804	0.374	Higher with MRF
Productivity	**Proportional laying days (%)**	**95.960**	**5.878**	**0.061**	**89.560**	**44.006**	**0.491**	**0.125**	**7.872**	**0.007**	**Lower with MRF**
	**Total egg count (*n*)**	**644.600**	**271.920**	**0.422**	**601.760**	**1969.622**	**3.273**	**0.129**	**7.601**	**0.008**	**Lower with MRF**
	**Total egg mass (Score)**	**235.720**	**65.562**	**0.278**	**216.520**	**227.770**	**1.052**	**0.264**	**5.012**	**0.030**	**Lower with MRF**
	**Total egg weight (kg/pen)**	**39.601**	**1.830**	**4.62*e***^**−05**^	**36.370**	**6.475**	**1.78*e***^**−04**^	**0.260**	**5.153**	**0.028**	**Lower with MRF**
Quality	Albumin height (mm)	4.858	0.620	0.128	4.828	0.852	0.177	0.723	0.490	0.857	Lower with MRF
	Haugh unit (unitless)	64.926	67.109	1.034	65.252	91.928	1.409	0.734	0.780	0.857	Lower with MRF
	Mean egg weight (g)	62.600	19.277	0.307	62.000	24.070	0.388	0.791	0.231	0.603	Lower with MRF
	Shell strength (kg/m^3^)	3.542	0.869	0.245	3.879	0.478	0.123	1.990	2.436	0.174	Higher with MRF
	Shell thickness (mm)	0.354	0.002	0.006	0.323	0.002	0.006	0.914	0.012	0.013	Lower with MRF
	Yolk colour (unitless)	2.880	0.266	0.092	3.120	0.346	0.111	0.833	0	0.157	No difference

Significant results (*P* < 0.05) are emboldened. Regarding statistical nomenclature, μ, σ^2^, *I*, ϙ, and *W* refer to mean, variance, index-of-dispersion, the qoppa relative index of dispersion, and Shapiro–Wilk test statistic, respectively

### Identification of potential quality and production factor impacting taxa

At the Phylum rank, Actinobacteria and *Ca*. Saccharibacteria were significantly negatively correlated with egg production, egg mass, and egg weight (Additional file 2: Table S21). Ignavibacteria reported significantly negative correlations with egg mass and egg weight. Thermotogae reported significant negative correlations with shell strength. Coprothermobacterota and Gemmatimonadetes were positively correlated with laying frequency. All of these taxa were significantly lower in MRF-supplemented eggs when compared using ANCOM (Additional file 2: Table S14). Shell strength was not significantly different in MRF-supplemented birds so the negative correlation with Thermotogae may suggest that Thermotogae does not impact shell strength and just correlates with taxa that do. The disparity observed between Coprothermobacterota and Gemmatimonadetes positive correlation with egg production, their lower abundance in MRF-supplemented birds, and the significantly greater egg production in MRF-supplemented birds may also suggest that these rare taxa do not impact productivity but correlate with another that does. The comparative rarity (η_%_ ≤ 0.0062%) and uneven intergroup compositional distributions of Coprothermobacterota, Gemmatimonadetes, and Thermotogae may also add weight to the argument that these taxa have little or no impact on quality and productivity factors.

**Fig. 8:**
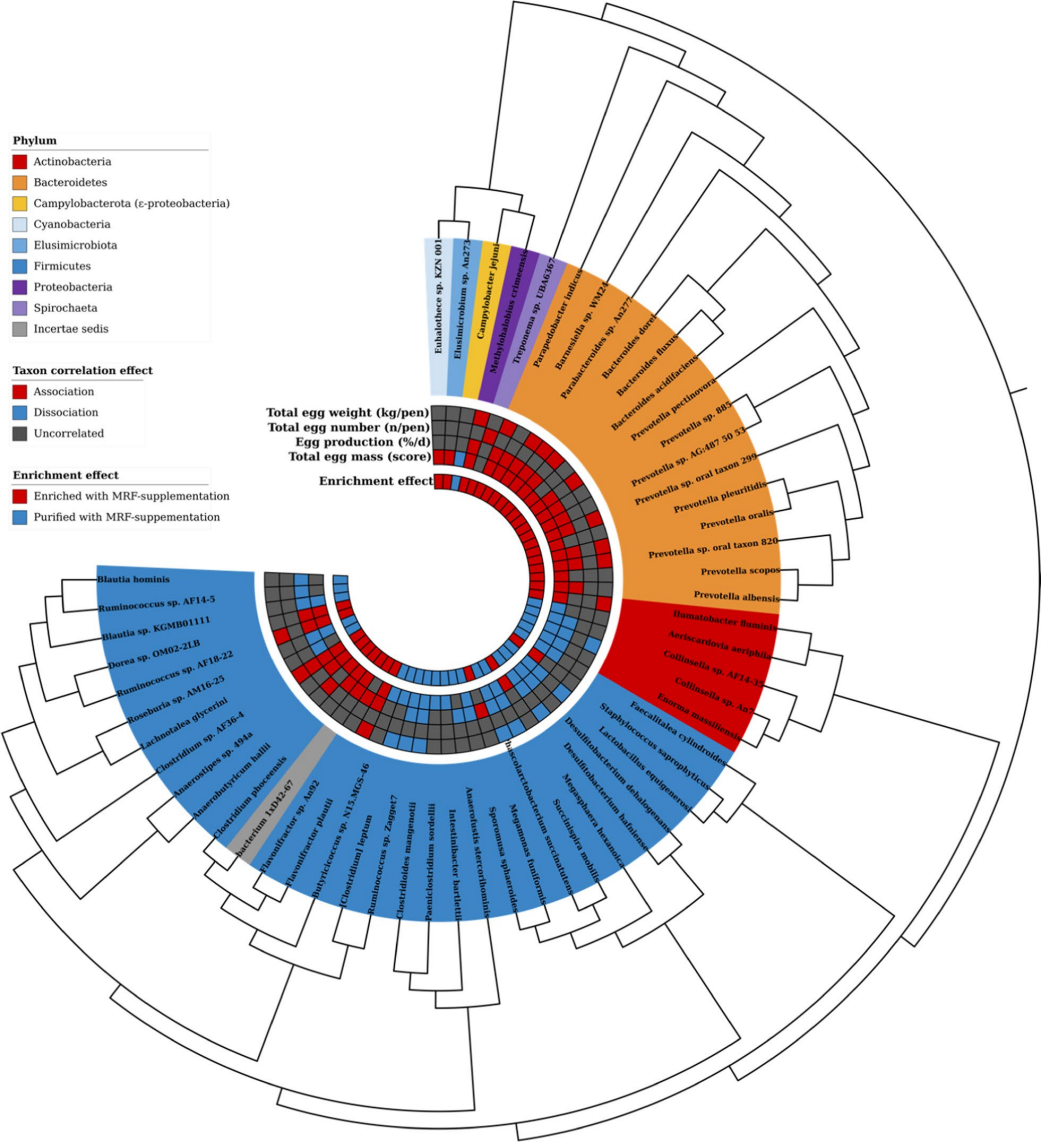
Phylogenetic distributions of significantly enriched taxa and their correlations with productivity factors

Feed conversion ratio was not correlated with any sampled species. All positive correlations stated in this section are statistically significant and associated with taxa that were significantly greater in MRF-supplemented birds (using ANCOM). At the species level rank, a total of 29 taxa were positively correlated with egg production (Fig. 8): one Actinobacteria (*Ilumatobacter fluminis*); 15 Bacteroidetes, of which, three were of the genus *Bacteroides* (*B. acidifaciens*, *B. dorei,* and *B. fluxus*); nine were of the genus *Prevotella* (*P. albensis*, *P. oralis*, *P. pectinovora*, *P. pleuritidis*, *P. scopos*, *Prevotella* sp. 885, *Prevotella* sp. AG:487 50 53, *Prevotella* sp. oral taxon 299, *Prevotella* sp. oral taxon 820; one of genus *Barnisella* (*Barnesiella* sp. WM24), one of genus *Parabacteroides* (*Parabacteroides* sp. An277), and one Sphingobacteria (*Parapedobacter indicus*); one Cyanobacteria (*Euhalothece* sp. KZN 001); one Elusimicrobium (*Elusimicrobium* sp. An273); nine Firmicutes, of which one was in class Bacilli (*Staphylococcus saprophyticus*) and one of class Negativicutes (*Megasphaera hexanoica*), the remaining seven were of class Clostridia, of which two were within the Clostridiaceae family (*Clostridium phoceensis* and *Clostridium* sp. AF36-4), three Lachnospiraceae (*Anaerobutyricum hallii*, *Anaerostipes* sp. 494a, and *Dorea* sp. OM02-2LB), and two of which were Ruminococcaceae (*Flavonifractor plautii* and *Ruminococcus* sp. AF18-22); one (Gamma-) Proteobacteria (*Methylohalobius crimeensis*); and bacterium 1xD42-67, a bacterial taxon of *incertae sedis* phylogenetic placement. In addition to egg productivity, 12 of the above taxa (*B. dorei*, *B. fluxus*, *P. oralis*, *P. pectinovora*, *Prevotella* sp. oral taxon 299, *Prevotella* sp. oral taxon 820, *Parabacteroides* sp. An277, *P. indicus*, *Clostridium* sp. AF36-4, *F. plautii*, *Ruminococcus* sp. AF18-22, and *M. crimeensis*) were also significantly positively correlated with total egg mass, total egg number, and total egg weight. In addition to egg productivity, eight taxa (*Barnesiella* sp. WM24*, P. albensis*, *Prevotella* sp. 885, *Prevotella* sp. AG:487 50 53, *C. phoceensis*, *Anaerostipes* sp. 494a, *Dorea* sp. OM02-2LB, and bacterium 1xD42-67) were significantly correlated with total egg mass and total egg weight but not total egg number. One taxon (*Ilumatobacter fluminis*) was positively correlated with egg production and egg number but not total egg mass or total egg weight. For completeness, eight taxa (*B. acidifaciens*, *P. pleuritidis*, *P. scopos*, *Euhalothece* sp. KZN 001, *Elusimicrobium* sp. An273, *S. saprophyticus*, *A. hallii*, and *M. hexanoica*) were positively correlated with egg production but not total egg weight, total egg mass, or total egg number. Finally, two Firmicute taxa (*Flavonifractor* sp. An92 and *Megamonas funiformis*) were positively correlated with total egg mass and total egg weight but not egg productivity or total egg number. Shell thickness was positively correlated with three taxa, two Firmicutes (*Clostridium* sp. AF36-4 (Firmicutes) and *Anaerobutyricum hallii*) and one Spirochaete (*Treponema* sp. UBA6367). Two Firmicute taxa were positively correlated with average egg weight: *Clostridium* sp. OF03-18AA and *Anaerostipes* sp. 494a.

All negative correlations stated in this section are all statistically significant and were associated with taxa that were significantly lower in MRF-supplemented birds (using ANCOM). A total of 20 taxa returned negative correlations with egg productivity. Of these, four were with the phylum Actinobacteria (*Aeriscardovia aeriphila*, *Collinsella* sp. AF14-35, *Collinsella* sp. An7, and *Enorma massiliensis*); 15 Firmicutes were observed, one within class Bacilli (*Lactobacillus equigenerosi*), one within class Erysipelotrichia (*Faecalitalea cylindroides*), and three within class Negativicutes (*Phascolarctobacterium succinatutens*, *Succinispira mobilis*, and *Sporomusa sphaeroides*); the remaining ten taxa were within class Clostridia (order Clostridiales), of which one was within family Clostridiaceae (*Butyricicoccus* sp. N15.MGS-46), two within family Lachnospiraceae (*Blautia* sp. KGMB01111 and Roseburia sp. AM16-25), two within family Peptococcaceae (*Desulfitobacterium dehalogenans* and *D. hafniense*), three within family Peptococcaceae (*Clostridioides mangenotii, Intestinibacter bartlettii*, *Paeniclostridium sordelli*), and two within family Ruminococcaceae ([Clostridium] *leptum* and *Ruminococcus* sp. Zagget7). Of those negatively correlated with egg productivity, a total of seven taxa (*Collinsella* sp. An7, *L. equigenerosi*, *D. hafniense*, *C. mangenotii, Ruminococcus* sp. Zagget7, *Phascolarctobacterium succinatutens*, and *Succinispira mobilis*) were also negatively correlated with shell thickness, total egg number, and total egg weight. Another seven taxa (*Collinsella* sp. AF14-35, *E. massiliensis*, *Blautia* sp. KGMB01111, *Roseburia* sp. AM16-25, *D. dehalogenans*, *F. cylindroides*, and *S. sphaeroides*) were negatively correlated with egg production, shell thickness and total egg weight but not total number. One taxon ([Clostridium] *leptum*) was also negatively correlated with total egg number but not total egg weight or shell thickness. One taxon (*C. jejuni*) was only negatively correlated with egg production but not total egg mass, total egg weight, or total egg number. Negative egg mass and negative total egg weight were always co-occurring. Four Firmicutes (Clostridales) taxa did not negatively correlate with egg production but did negatively correlate with total egg weight and total egg mass: *Anaerofustis stercorihominis*, *Blautia hominis*, *Lachnotalea glycerini*, *Ruminococcus* sp. AF14-5.

## Discussion

As the commercial layer diet is comprised of approximately 70% grain, maximal feed efficiency from these sources is of paramount economic importance [39]; as such the significantly better FCE observed following MRF-supplementation should aid in egg farm economic sustainability. Chickens lack the capacity to digest fibre (*e.g.* arabinoxylan and (1,3;1,4)-β-glucan) and rely on complex cecal microbiota to extract energy and nutrients from these sources [9, 41, 53]. An indicator of increased cecal fibre digestion is an increase in SCFA producer species [86, 94]. In this study, we observed greater abundances in butyrate, acetate and propionate producers (*e.g. Anaerobutyricum hallii*, *Anaerotignum neopropionicum, Butyricoccus porcorum, Bacteroides acidifasciens, Alistipes shahii,* and *B. ovatus*).

Flock uniformity is also important for economic return-on-investment in agriculture [61]. Reduced flock uniformity may translate to decreased profitability due to variations in achieving optimal production traits and market specifications. The composition and activity of the gut microbiome is predominantly shaped by dietary, management, and environmental factors and is known to impact animal health and productivity. As such, a more uniform microbiota composition may be an indicator of gut stability (and flock uniformity) by limiting the effect, and progression of dysbiosis which would have negative health and productivity consequences [44, 65, 84].

The significant changes in productivity factors observed in this study were particularly striking. As average egg weights were not statistically different and as each bird was estimated to lay single eggs, the greater total egg weight, numbers, and mass (6.79%-7.42%) are most likely due to the 7.78% higher laying frequency which represents an additional 2–3 eggs per bird (2.36 eggs) in a 28-day period or 14.26 eggs per bird over a 169-day period. These greater productivity observations were observed alongside a significantly (5.58%) better FCR without a statistical difference in feed intake or weight differences in birds. Furthermore, the four productivity factors also displayed significantly less dispersion following MRF supplementation, representing a 4–eightfold reduction in dispersion. While MRF supplementation resulted in greater and more uniform productivity factors, quality factors were statistically unaffected. These results highlight the economic potential of MRF-supplementation for productivity and consistency without a detectable impact on product quality.

When the correlations between taxa and productivity factors were explored, differential patterns were observed despite the likelihood that total egg weight, number, and mass are derived from higher laying frequency. When taxa that significantly associated or dissociated with all four factors were considered, some interesting patterns were observed. In total, 12 taxa were positively associated with productivity and seven were negatively associated. Of the positively associated taxa, eight were Bacteroidetes (two from genus *Bacteroides* (*B. dorei* and *B. fluxus*), four from genus *Prevotella* (*P. oralis*, *P. pectinovora*, *Prevotella* sp. oral taxon 299, and *Prevotella* sp. oral taxon 820), *Parabacteroides* sp. An277*,* and *Parapedobacter indicus*), three Clostridia (*Clostridium* sp. AF36-4, *Flavonifractor plautii*, *Ruminococcus* sp. AF18-22) and one gammaproteobacterium (*Methylohalobius crimeensis*). Follicle-stimulating hormone (FSH) and leutinising hormone (LH) concentrations are strongly associated with laying frequency in hens, where both of these hormones are influenced by estradiol concentrations [71, 85]. In several human and animal studies, *Prevotella* and *Bacteroides* abundances are correlated with estradiol [48, 82, 106], and estradiol has been observed promoting *Bacteroides* growth [45]. Several *Prevotella* and *Bacteroides* species produce β-glucuronidase which is capable of deconjugating estradiol-17-glucuronide back to estrodiol and, therefore, may influence estrodiol coordinated LH and FSH release [28, 82]. While we observed both significantly greater abundances in the genus *Bacteroides*, several *Bacteroides* and *Prevotella* species, and laying frequency, we did not conduct any endocrinological analyses on the hens so further investigation is required to confirm whether this interaction is responsible for increased lay.

When the effect on MRF-supplementation on individual taxa was investigated, the overall phylum level Firmicutes abundances were significantly lower, however, 17 Firmicutes species were significantly greater. While this trend may seem counterintuitive, all these species (with the exception of *Megamonas funiformis*) are relatively rare (η% < 1%). Interestingly, Proteobacteria were not significantly lower with MRF-supplementation (as is often observed in broiler studies [18, 22, 53, 98, 100]. Proteobacteria in both control and MRF-supplemented birds were rare (η% < 1%) which may indicate population stability at this timepoint in the layer lifecycle. Higher compositional proportions of *C. jejuni* are associated with the development of arthritis in hens and jejunal distention, disseminated haemorrhagic enteritis, and focal hepatic necrosis in chicks [3, 95]. Foodborne pathogens, such as *C. jejuni* may pass into the food chain via both chicken meat and, albeit much more rarely, egg products [32, 34] so any bioburden reduction of these species is of importance from a foodchain integrity perspective.

In this study, α-diversity metrics were considerably affected in the MRF-supplemented birds when compared to the control. A particularly striking observation was a negative correlation between taxonomic richness and taxonomic evenness (where significantly greater richness and significantly lower evenness was observed in MRF-treated birds). Interestingly, greater effects were observed in lower ranks than in higher ranks, suggesting that while lower taxonomic ranks were dynamic, higher ranks were more stable. In addition to α-diversity metrics, β-diversity were also observed to be significantly different between treatment groups indicating compositional heterogeneity differences [10, 52, 101]. In this study, at every taxonomic rank, MRF-supplemented and control groups were observed to be significantly separated and were observed to have significantly greater intergroup differences than intragroup differences. These results suggest that while MRF-supplemented and control groups considerably differ, the underlying communities follow similar compositional patterns, suggesting community-wide treatment effect rather than a unique effect. These results are consistent with the significant impact on several high abundance microbiota but lack of displacement from the 20 most abundant taxa.

Cecal microbiota composition and perturbation is a key determinant of performance and health status in livestock [61, 91, 108]. In previous studies, increased intestinal microbiota diversity has been strongly correlated with pathogen colonisation resistance, dietary energy extraction efficiency, amino acid biosynthesis, vitamin biosynthesis, and short chain fatty acid biosynthesis [9, 53, 58]. Both biotic (*e.g.* infection) and abiotic (*e.g.* temperature change) factors can lead to dysbiosis, yielding decreased diversity and subsequent decreases in this health status and performance. While this concept has been the subject of extensive study in broilers [18, 22, 53], relatively few studies, to our knowledge, exist for mature layers. In this study, MRF-supplemented birds established greater α- and β-diversity metrics in post-peak laying hens, greater compositional uniformity across samples, a lower pathogenic bioburden, and a greater abundance of correlators of performance.

## Conclusion

This large study demonstrated that MRF-supplementation of layer hens yielded richer, more uniform cecal microbiota communities, better populated with health promoting commensal species within the caeca. Supplementation with MRF has previously been shown to result in greater taxonomic richness and altered microbiota in broilers [22, 53]. The significantly lower abundance of *C. jejuni,* in alignment with previous broiler studies, yields greater food chain integrity. Overall, these results suggest that MRF-supplementation has a role in promoting a healthy microbiota and reducing dysbiosis. Effective gastrointestinal functionality is crucial in supporting animal health, welfare, and performance.

### Supplementary Information

**Additional file 1**. SI Figures.

**Additional file 2**. SI Tables.

## Data Availability

16S rRNA reads associated with this study are available at NCBI BioProject PRJNA1027632 (https://www.ncbi.nlm.nih.gov/bioproject/). Reads are publicly available from the date of publication.

## Abbreviations:

16S rRNA: 16S Svedbard ribosomal ribonucleic acid
2D/3D: 2 Dimensional/3 dimensional
ADFI: Average daily feed intake
ANCOM: Analysis of composition of microbiomes
ANOSIM: Analysis of similarity
BW: Body weight
EP: Egg production
EW: Egg weight
MRF: Mannan rich fraction
*n*_*x*_: Number/count of *x*
PCA: Principal component analysis
PCoA: Principal coordinate analysis
PERMANOVA: Permutational analysis of variance
SI: Supplementary information
SFCA: Short chain fatty acid
Subsp.: Subspecies
*v.*: Version

## List of statistical abbreviations

μ: Mean
σ: Standard deviation
σ^2^: Variance
η: Median
~: Approximal to
≁: Not approximal to
*E*: Expected value
FC: Fold change
G: Centroid
H_0_: Null hypothesis
H_A_: Alternative hypothesis
ln: Natural log
*N*(μ,σ^2^): Normal (Gaussian) distribution
*P*: *P*-Value
*R*: Rank
*X*: Sample distribution

## Statistical null hypotheses

ANCOM: *E*[Ln(µ_*x*_^(^^*a*^^)^)] = *E*[ln(µ_*x*_^(^^*b*^^)^)] The expected value for the natural log of the mean of taxon* x* is equivalent for treatment groups *a* and *b*.
ANOSIM: *R* = 0 Rank resemblances overlap (similarity between treatment groups ≥ similaritywithin respective groups)
Brunner-Munzel test: *B* = 0.5 Both treatment groups are stochastically equivalent
Fisher’s exact test: *p̂*_*a*_ = *p̂*_*b*_ The proportionality of subpopulation *p* is equal between populations *a* and *b*
Levene’s test: σ^2^_*x*_^(^^*a*^^)^ = σ^2^_*x*_^(^^*b*^^)^ The variance for taxon *x* is equivalent for treatment groups *a* and *b*.
PERMANOVA: G_(__*a*__)_ ~ G_(__*b*__)_ Centroid positions are approximal between treatment groups *a* and *b*
Shapiro–Wilk test: *X*_*x*_ ~ *N*(μ,σ^2^) The sample distribution for taxon *x* a approximates a Gaussian distribution

## References

[1] Aitchison J (1982). The statistical analysis of compositional data. J Roy Stat Soc: Ser B (Methodol).

[2] Al-Khalaifa H (2019). Effect of dietary probiotics and prebiotics on the performance of broiler chickens. Poult Sci.

[3] Alpigiani I (2017). Associations between animal welfare indicators and Campylobacter spp. in broiler chickens under commercial settings: A case study. Prev Vet Med.

[4] Anderson MJ (2001). A new method for non-parametric multivariate analysis of variance. Austral Ecol.

[5] Andrews S et al. (2015) ‘FastQC. A quality control tool for high throughput sequence data. Babraham Bioinformatics’, 1(1), p. undefined-undefined. Available at: https://www.mendeley.com/catalogue/8057171e-e700-36a0-b936-1c307058462d/?utm_source=desktop&utm_medium=1.19.8&utm_campaign=open_catalog&userDocumentId=%7B124edc8f-5459-3c1b-a449-8ab9bc3e95f8%7D (Accessed: 18 August 2021).

[6] Anene DO (2020). Variation and association of hen performance and egg quality traits in individual early-laying ISA brown hens. Animals.

[7] Anene DO (2023). Effect of restricted feeding on hen performance, egg quality and organ characteristics of individual laying hens. Animal Nutrition.

[8] Benjamin DJ (2018). Redefine statistical significance. Nat Hum Behav.

[9] Borey M (2020). Broilers divergently selected for digestibility differ for their digestive microbial ecosystems. PLoS ONE.

[10] Bray JR, Curtis JT (1957). An ordination of the upland forest communities of southern Wisconsin. Ecol Monogr.

[11] Brunner E, Munzel U (2000). The nonparametric Behrens–Fisher problem: asymptotic theory and a small-sample approximation. Biom J.

[12] Capella-Gutierrez S, Silla-Martinez JM, Gabaldon T (2009). trimAl: a tool for automated alignment trimming in large-scale phylogenetic analyses. Bioinformatics.

[13] Chacher MFA (2017). Use of mannan oligosaccharide in broiler diets: an overview of underlying mechanisms. World’s Poultry Science Journal.

[14] Chao A (1984). Nonparametric Estimation of the Number of Classes in a Population. Scand J Stat.

[15] Clarke KR (1993). Non-parametric multivariate analyses of changes in community structure. Austral Ecol.

[16] Cole MB (2018). The science of food security. Sci Food.

[17] Coleman GA (2021). A rooted phylogeny resolves early bacterial evolution. Science.

[18] Corrigan A (2018). The use of random forests modelling to detect yeast-mannan sensitive bacterial changes in the broiler cecum. Sci Rep.

[19] Corrigan A (2023). Microbial community diversity and structure in the cecum of laying hens with and without mannan-rich fraction supplementation. J Appl Poul Res.

[20] Corrigan A, Corcionivoschi N, Murphy RA (2017). Effect of yeast mannan-rich fractions on reducing Campylobacter colonization in broiler chickens. J Appl Poultry Res.

[21] Dale N (1994). National research council nutrient requirements of poultry – ninth revised edition. J Appl Poul Res.

[22] Delaney S (2019). Microbiome and resistome of the gastrointestinal tract of broiler chickens. Access Microbiol.

[23] Denissen J (2022). Prevalence of ESKAPE pathogens in the environment: antibiotic resistance status, community-acquired infection and risk to human health. Int J Hyg Environ Health.

[24] Van Dongen S (2008). Graph clustering via a discrete uncoupling process. SIAM J Matr Anal Appl.

[25] Donoghue DJ (2003). Antibiotic residues in poultry tissues and eggs: human health concerns?. Poult Sci.

[26] Du MZ (2018). The GC content as a main factor shaping the amino acid usage during bacterial evolution process. Front Microbiol.

[27] Edgar RC (2004). MUSCLE: multiple sequence alignment with high accuracy and high throughput. Nucleic Acids Res.

[28] Ervin SM (2019). Gut microbial-glucuronidases reactivate estrogens as components of the estrobolome that reactivate estrogens. J Biol Chem.

[29] Ester M et al. (1996) A density-based algorithm for discovering clusters in large spatial databases with noise

[30] Feng X (2022). Effects of challenge with Clostridium perfringens, Eimeria and both on ileal microbiota of yellow feather broilers. Front Microbiol.

[31] Fisher RA (1922). On the interpretation of χ 2 from contingency tables, and the calculation of P. J Roy Stat Soc.

[32] Fonseca BB (2014). Campylobacter jejuni in commercial eggs. Braz J Microbiol.

[33] Gao P (2017). Feed-additive probiotics accelerate yet antibiotics delay intestinal microbiota maturation in broiler chicken. Microbiome.

[34] Gharbi M (2022). Campylobacter spp. in eggs and laying hens in the north-east of Tunisia: high prevalence and multidrug-resistance phenotypes. Veterinary Sciences.

[35] Gloor GB (2017). Microbiome datasets are compositional: And this is not optional. Front Microbiol.

[36] Gruber-Vodicka HR, Seah BKB, Pruesse E (2020). phyloFlash: rapid small-subunit rRNA profiling and targeted assembly from metagenomes. mSystems.

[37] Hafez HM, Attia YA (2020). Challenges to the poultry industry: current perspectives and strategic future after the COVID-19 outbreak. Front Veter Sci.

[38] Hellinger E (1909). Neue Begründung der Theorie quadratischer Formen von unendlichvielen Veränderlichen’. J Reine Angew Math.

[39] Hooge DM, Kiers A, Connolly A (2013). Meta-analysis summary of broiler chicken trials with dietary actigen™ (2009–2012). Int J Poult Sci.

[40] Jaccard P (1912). The distribution of flora in the Alpine zone. New Phytol.

[41] Józefiak D, Rutkowski A, Martin SA (2004). Carbohydrate fermentation in the avian ceca: a review. Animal Feed Sci Technol.

[42] Jumas-Bilak E, Roudière L, Marchandin H (2009). Despcription of “Synergistetes” phyl. nov. and emended description of the phylum “Deferribacteres” and of the family Syntrophomonadaceae, phylum “Firmicutes”. Int J Syst Evol Microbiol.

[43] Kitts PA (2016). Assembly: a resource for assembled genomes at NCBI. Nucleic Acids Res.

[44] Kogut MH (2019). The effect of microbiome modulation on the intestinal health of poultry. Anim Feed Sci Technol.

[45] Kornman KS, Loesche WJ (1982). Effects of estradiol and progesterone on Bacteroides melaninogenicus and Bacteroides gingivalis. Infect Immun.

[46] Krueger F (2012) Babraham Bioinformatics - Trim Galore*!* Available at: https://www.bioinformatics.babraham.ac.uk/projects/trim_galore/ (Accessed: 16 January 2021)

[47] Kück P, Meusemann K (2010). FASconCAT: convenient handling of data matrices. Mol Phylogenet Evol.

[48] Kwa M (2016). The intestinal microbiome and estrogen receptor-positive female breast cancer. JNCI J National Cancer Inst.

[49] Landers TF (2012). A review of antibiotic use in food animals: perspective, policy, and potential. Public Health Rep.

[50] Lanthier M (2001). Geographic distribution of Desulfitobacterium frappieri PCP-1 and Desulfitobacterium spp. in soils from the province of QueBec, Canada. FEMS Microbiol Ecol.

[51] Le SQ, Gascuel O (2008). An improved general amino acid replacement matrix. Mol Biol Evol.

[52] Lee HJ (2018). Effects of cosmetics on the skin microbiome of facial cheeks with different hydration levels. MicrobiologyOpen.

[53] Leigh RJ (2022). Effect of Mannan-rich fraction supplementation on commercial broiler intestinum tenue and cecum microbiota. Animal Microbiome.

[54] Leigh RJ, Murphy R, Walsh F (2021) Uniforest: an unsupervised machine learning technique to detect outliers and restrict variance in microbiome studies. *bioRxiv* doi: 10.1101/2021.05.17.444491.

[55] Leigh RJ, Murphy RA, Walsh F (2021) statSuma: automated selection and performance of statistical comparisons for microbiome studies. *bioRxiv*. doi: 10.1101/2021.06.15.448299.

[56] Letunic I, Bork P (2021). Interactive tree of life (iTOL) v5: an online tool for phylogenetic tree display and annotation. Nucleic Acids Res.

[57] Levene H, Olkin I (1960). Robust Tests for Equality of Variances. Contributions to Probability and Statistics.

[58] Liao X (2020). The relationship among gut microbiota, short-chain fatty acids, and intestinal morphology of growing and healthy broilers. Poult Sci.

[59] Liu C (2008). Reclassification of Clostridium coccoides, Ruminococcus hansenii, Ruminococcus hydrogenotrophicus, Ruminococcus luti, Ruminococcus productus and Ruminococcus schinkii as Blautia coccoides gen. nov., comb. nov., Blautia hansenii comb. nov., Blautia hydrogenotrophica comb. nov., Blautia luti comb. nov., Blautia producta comb. nov., Blautia schinkii comb. nov. and description of Blautia wexlerae sp. nov., isolated from human faeces. Int J Syst Evol Microbiol.

[60] Liu X (2021). Blautia-a new functional genus with potential probiotic properties?. Gut microbes.

[61] Lundberg R, Scharch C, Sandvang D (2021). The link between broiler flock heterogeneity and cecal microbiome composition. Animal Microbiome.

[62] Luquetti BC (2012). Saccharomyces Cerevisiae cell wall dietary supplementation on the performance and intestinal mucosa development and integrity of broiler chickens vaccinated against coccidiosis. Brazil J Poul Sci.

[63] Ma F (2021). Use of antimicrobials in food animals and impact of transmission of antimicrobial resistance on humans. Biosafety and Health.

[64] Mak PHW (2022). Production systems and important antimicrobial resistant-pathogenic bacteria in poultry: a review. J Anim Sci Biotechnol.

[65] Mancabelli L (2016). Insights into the biodiversity of the gut microbiota of broiler chickens. Environ Microbiol.

[66] Mandal S (2015). Analysis of composition of microbiomes: a novel method for studying microbial composition. Microbial Ecol Health Dis.

[67] Martín-Fernández JA, Barceló-Vidal C, Pawlowsky-Glahn V (2003). Dealing with zeros and missing values in compositional data sets using nonparametric imputation. Math Geol.

[68] Martin M (2011). Cutadapt removes adapter sequences from high-throughput sequencing reads. EMBnetjournal.

[69] McCaffrey C (2021). Effect of yeast cell wall supplementation on intestinal integrity, digestive enzyme activity and immune traits of broilers. Br Poult Sci.

[70] Medvecky M (2018). Whole genome sequencing and function prediction of 133 gut anaerobes isolated from chicken caecum in pure cultures. BMC Genomics.

[71] Mehlhorn J (2022). Estradiol-17ß Is influenced by age, housing system, and laying performance in genetically divergent laying Hens (Gallus gallus fd). Front Physiol.

[72] More SJ (2020). European perspectives on efforts to reduce antimicrobial usage in food animal production. Ir Vet J.

[73] Morris EK (2014). Choosing and using diversity indices: insights for ecological applications from the German Biodiversity Exploratories. Ecol Evol.

[74] Nguyen L-T (2015). IQ-TREE: a fast and effective stochastic algorithm for estimating maximum-likelihood phylogenies. Mol Biol Evolut.

[75] Oakley BB (2014). The chicken gastrointestinal microbiome. FEMS Microbiol Lett.

[76] De Oliveira DMP (2020). Antimicrobial resistance in ESKAPE pathogens. Clin Microbiol Rev.

[77] Oren A, Garrity GM (2021). Valid publication of the names of forty-two phyla of prokaryotes. Int J Syst Evolut Microbiol.

[78] Pandit RJ (2018). Microbial diversity and community composition of caecal microbiota in commercial and indigenous Indian chickens determined using 16s rDNA amplicon sequencing. Microbiome.

[79] Patil A (2021). Foodborne ESKAPE biofilms and antimicrobial resistance: lessons learned from clinical isolates. Pathogens and Global Health.

[80] Pearson K (1901). LIII. On lines and planes of closest fit to systems of points in space. The London, Edinburgh, and Dublin Philosophical Magazine and Journal of Science.

[81] Pedregosa F et al*.* (2011) Scikit-learn: Machine Learning in Python Gaël Varoquaux Bertrand Thirion Vincent Dubourg Alexandre Passos PEDREGOSA, VAROQUAUX, GRAMFORT ET AL. Matthieu Perrot, Journal of Machine Learning Research. Available at: http://scikit-learn.sourceforge.net. (Accessed: 16 January 2021)

[82] Peters BA (2022). ‘Menopause is associated with an altered gut microbiome and estrobolome, with implications for adverse cardiometabolic risk in the hispanic community health study/study of latinos. mSystems.

[83] Pielou EC (1966). The measurement of diversity in different types of biological collections. J Theor Biol.

[84] Pourabedin M, Zhao X (2015). Prebiotics and gut microbiota in chickens. FEMS Microbiol Lett.

[85] Prastiya RA (2022). Effect of follicle-stimulating hormone and luteinizing hormone levels on egg-laying frequency in hens. Veterin World.

[86] Rebolé A (2010). Effects of inulin and enzyme complex, individually or in combination, on growth performance, intestinal microflora, cecal fermentation characteristics, and jejunal histomorphology in broiler chickens fed a wheat- and barley-based diet. Poult Sci.

[87] Rosenberg E (2007). The role of microorganisms in coral health, disease and evolution. Nature Rev Microbiol.

[88] Rosenberg E, Zilber-Rosenberg I (2016). ‘Microbes drive evolution of animals and plants: The hologenome concept’, *mBio*. American Society for Microbiology.

[89] Roswell M, Dushoff J, Winfree R (2021). A conceptual guide to measuring species diversity. Oikos.

[90] Rychlik I (2020). Composition and Function of Chicken Gut Microbiota. Animals : an open access journal from MDPI.

[91] Salaheen S (2017). Alternative growth promoters Modulate broiler gut microbiome and enhance body weight gain. Front Microbiol.

[92] Salami SA (2022). Performance and environmental impact of egg production in response to dietary supplementation of mannan oligosaccharide in laying hens: a meta-analysis. Poul Sci.

[93] Seemann T (2014). Prokka: Rapid prokaryotic genome annotation. Bioinformatics.

[94] Sergeant MJ (2014). Extensive microbial and functional diversity within the chicken cecal microbiome. PLoS ONE.

[95] Shane SM (1992). The significance of campylobacter jejuni infection in poultry: a review. Avian Pathol.

[96] Shannon CE (1948). A mathematical theory of communication. Bell Syst Tech J.

[97] Simpson EH (1949). Measurement of diversity. Nature.

[98] Smith H (2020). Yeast cell wall mannan rich fraction modulates bacterial cellular respiration potentiating antibiotic efficacy. Sci Rep.

[99] Spring P (2000). The effects of dietary mannaoligosaccharides on cecal parameters and the concentrations of enteric bacteria in the ceca of salmonella-challenged broiler chicks. Poult Sci.

[100] Spring P (2015). A review of 733 published trials on Bio-Mos®, a mannan oligosaccharide, and Actigen®, a second generation mannose rich fraction, on farm and companion animals. J Appl Anim Nutr.

[101] Stanley D (2016). Bacteria within the gastrointestinal tract microbiota correlated with improved growth and feed conversion: challenges presented for the identification of performance enhancing probiotic bacteria. Front Microbiol.

[102] Thanner S, Drissner D, Walsh F (2016). Antimicrobial resistance in agriculture. MBio.

[103] Tilman D (2011). Global food demand and the sustainable intensification of agriculture. Proc Natl Acad Sci.

[104] Villemur R (2006). The Desulfitobacterium genus. FEMS Microbiol Rev.

[105] Waite DW (2017). Comparative genomic analysis of the class Epsilonproteobacteria and proposed reclassification to epsilonbacteraeota (phyl. nov.). Front Microbiol.

[106] Wang Z (2021). An emerging role of Prevotella histicola on estrogen deficiency-induced bone loss through the gut microbiota-bone axis in postmenopausal women and in ovariectomized mice. Am J Clin Nutr.

[107] Xiao Y (2017). Microbial community mapping in intestinal tract of broiler chicken. Poult Sci.

[108] Zhang S (2021). Dietary supplementation with Bacillus subtilis promotes growth performance of broilers by altering the dominant microbial community. Poult Sci.

