# Correction: Yeast mannan rich fraction positively influences microbiome uniformity, productivity associated taxa, and lay performance

**DOI:** 10.1186/s42523-024-00310-x

**Published:** 2024-05-02

**Authors:** Robert J. Leigh, Aoife Corrigan, Richard A. Murphy, Jules Taylor-Pickard, Colm A. Moran, Fiona Walsh

**Affiliations:** 1 Department of Biology, Maynooth University, Maynooth, Co. Kildare Ireland; 2 Alltech Bioscience Centre, Dunboyne, Co. Meath Ireland; 3 Alltech (UK) Ltd., PE9 1TZ Stamford, UK; 4 Alltech SARL, Rue Charles Amand, 14500 Vire, France

**Correction to: Animal Microbiome**10.1186/s42523-024-00295-7.

Following publication of the original article [1], we have been requested to add 2 authors to the authorship group.

**It is now as follows:**

Robert J. Leigh1* †, Aoife Corrigan2†, Richard A. Murphy2 and Fiona Walsh1*

**It should be as follows:**

Robert J. Leigh1* †, Aoife Corrigan2†, Richard A. Murphy2, Jules Taylor-Pickard3, Colm A. Moran 4 and Fiona Walsh1*

Also, Acknowledgement section, Author contributions and Competing interests’ sections need to be updated.

**Acknowledgement section now is as follows**:

N.A.

**Acknowledgement section should be as follows**:

The authors would like to thank Doug Currie and Dr. Lauren Park of Roslin Nutrition Ltd. (Aberlady, Scotland) for conducting the layer study and formulating and preparing the layer diets. Our gratitude to Dr. Jason Keegan (Alltech Bioscience Centre (Ireland)) for project administration (animal science component) and initial data analysis (animal science component).

**Author contributions’ section now is as follows**:

RL formatted all data for downstream analyses, performed all statistical, computational biology, and bioinformatic analyses, applied computational approaches for measuring uniformity, devised the ϙ metric for measuring relative dispersion in productivity observations, produced all figures, formatted all tables, wrote the draft papers, and applied corrections as advised by other authors. AC coordinated sample collection, sequencing, and productivity observations. RM and FW provided research supervision and project direction.

All authors provided input to the corrected draft paper. The graphical abstract was produced under a paid licence with BioRender.com (2213–9170).

**Author contributions’ section should be as follows**:

RL formatted all data for downstream analyses, performed all statistical, computational biology, and bioinformatic analyses, applied computational approaches for measuring uniformity, devised the ϙ metric for measuring relative dispersion in productivity observations, produced all figures, formatted all tables, wrote the draft papers, and applied corrections as advised by other authors. AC coordinated sample collection, sequencing, and productivity observations. RM and FW provided research supervision and project direction.

All authors provided input to the corrected draft paper. The graphical abstract was produced under a paid licence with BioRender.com (2213–9170). JTP– Conceptualisation (animal science component), methodology (animal science component), project supervision (animal science component).

CM– Conceptualisation (animal science component), methodology (animal science component), project supervision (animal science component), funding acquisition (animal science component).

**Competing interests’ section now is as follows**:

RL was in receipt of a Postdoctoral Fellowship funded by Alltech for the duration of this study. AC and RM were in receipt of salaries from Alltech for the duration of this study. Alltech is a manufacturer of animal feed and dietary supplements.

**Competing interests’ section should be as follows**:

RL was in receipt of a Postdoctoral Fellowship funded by Alltech for the duration of this study. AC and RM were in receipt of salaries from Alltech for the duration of this study. Alltech is a manufacturer of animal feed and dietary supplements. CM and JTP are employees of Alltech which produces and markets Actigen®, the commercial product evaluated in this study.

The original article was updated.
