# Supplementary material for: Yeast mannan rich fraction positively influences microbiome uniformity, productivity associated taxa, and lay performance
Source: Anim Microbiome. 2024 Mar 4;6:9. doi: 10.1186/s42523-024-00295-7 (PMC10913240; doi:10.1186/s42523-024-00295-7)
Supplement: Supplementary file 1 — Additional file 1. SI Figures. [file 42523_2024_295_MOESM1_ESM.docx]

**
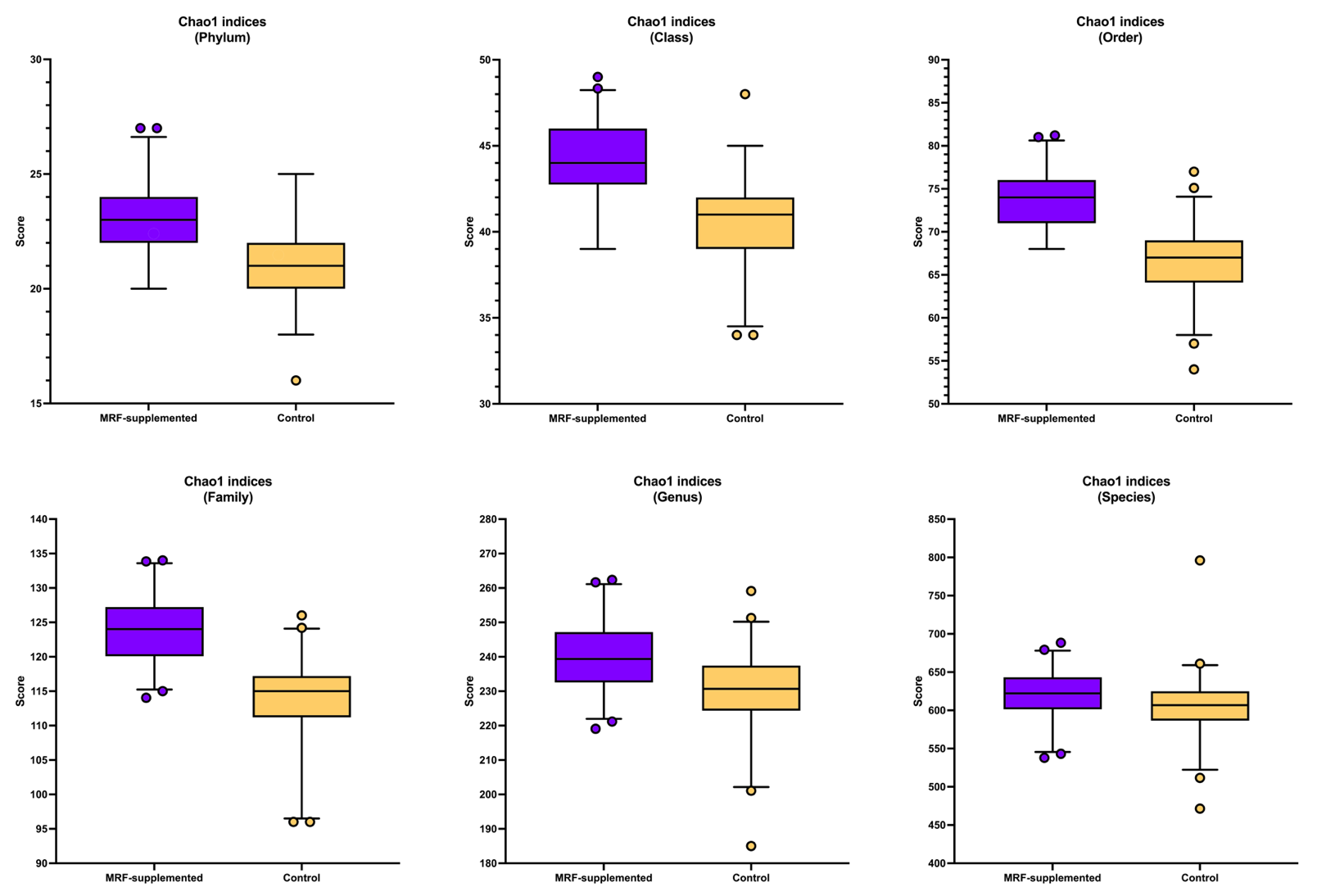
**

**SI Figure 1: Impact of MRF-supplementation on Chao1 indices (α-diversity)**

The horizontal line within each box denotes the mean and tails represent 95% confidence intervals. No observations exceeded the 95% CI intervals. In each plot *B* and *P* refer to the Brunner-Munzel test statistic and its associated *P*-value.

**
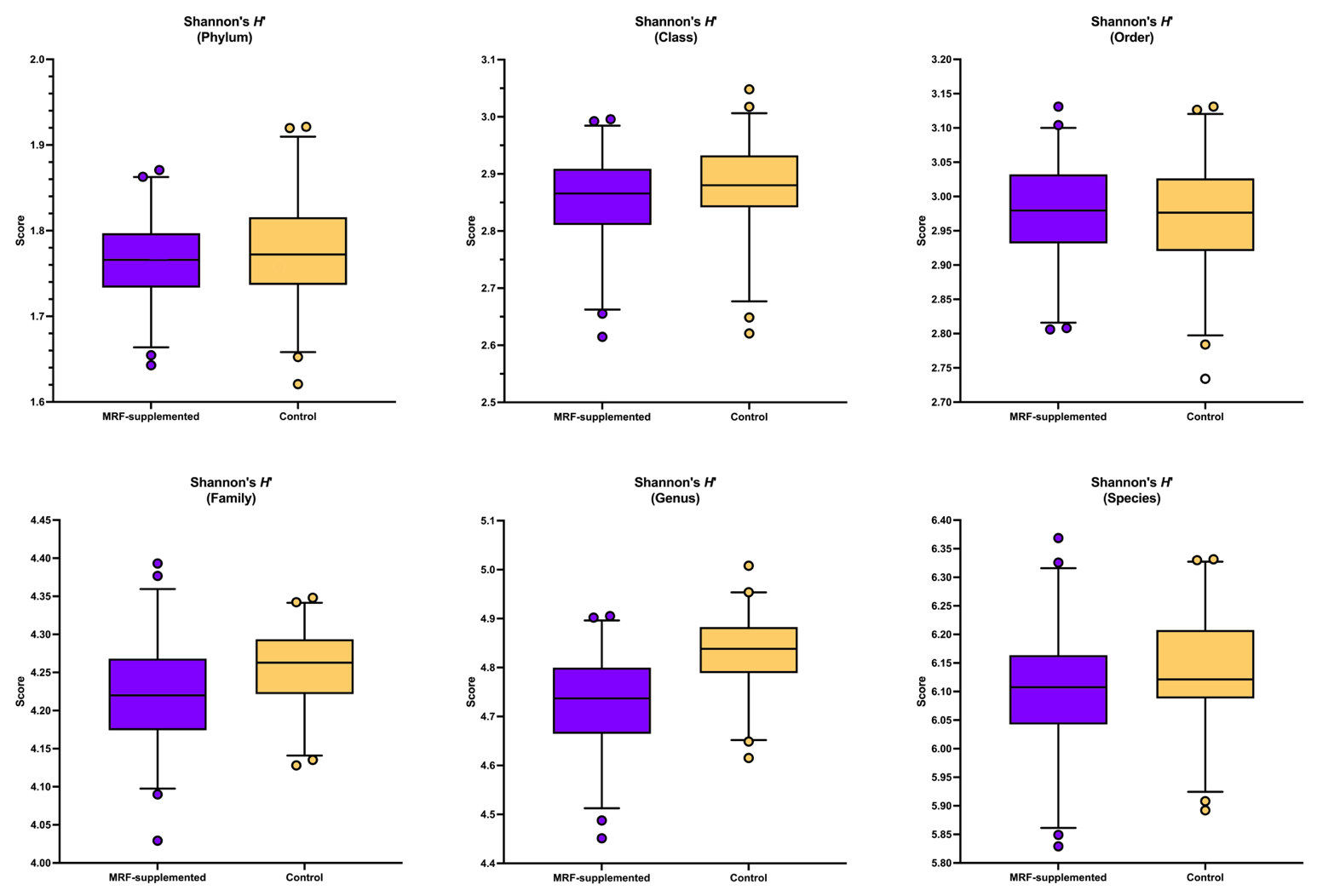
**

**SI Figure 2: Impact of MRF-supplementation on Shannon’s *H*’ (α-diversity)**

The horizontal line within each box denotes the mean and tails represent 95% confidence intervals. No observations exceeded the 95% CI intervals. In each plot *B* and *P* refer to the Brunner-Munzel test statistic and its associated *P*-value.

**
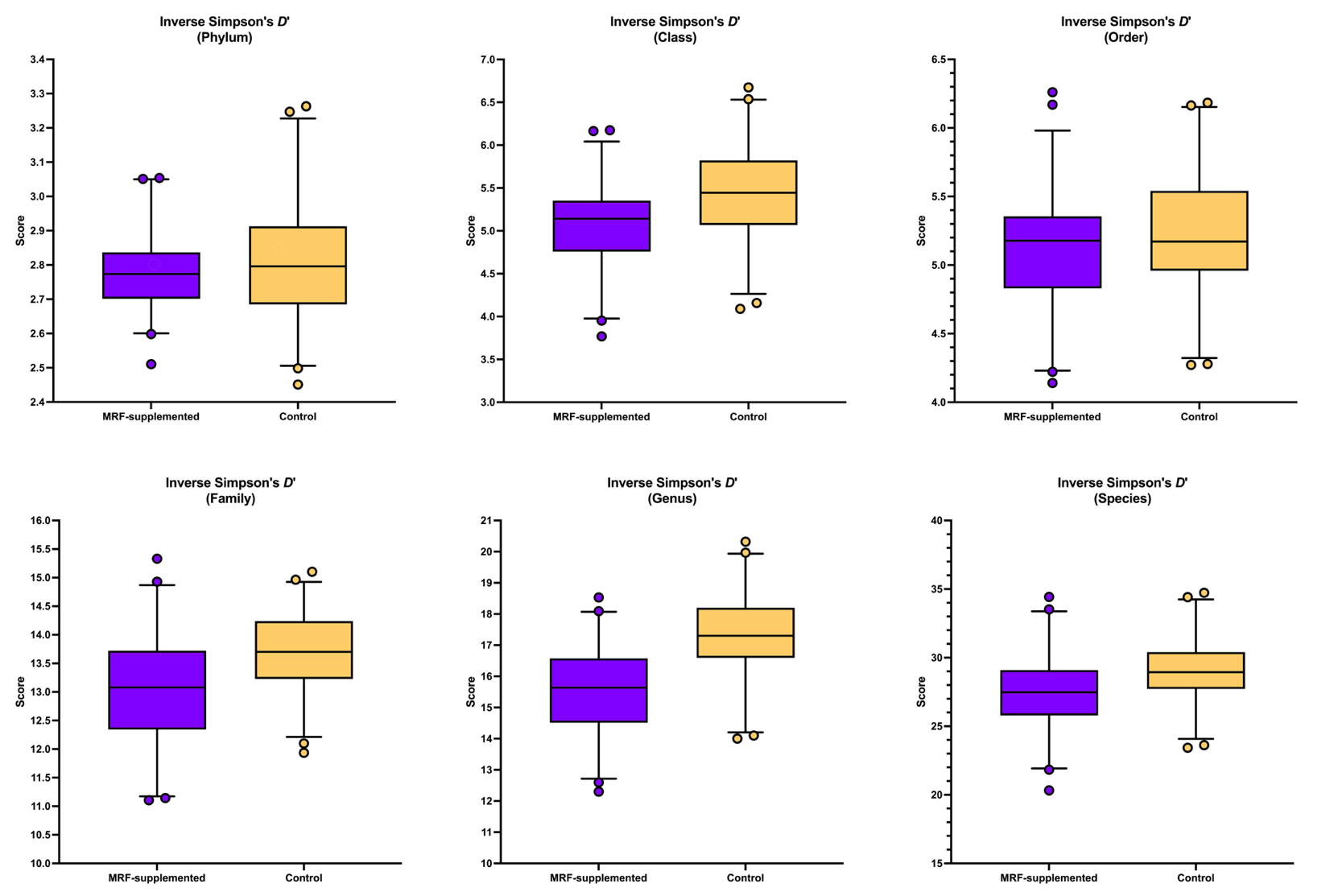
**

**SI Figure 3: Impact of MRF-supplementation on Inverse Simpson’s *D*’ (α-diversity)**

The horizontal line within each box denotes the mean and tails represent 95% confidence intervals. No observations exceeded the 95% CI intervals. In each plot *B* and *P* refer to the Brunner-Munzel test statistic and its associated *P*-value.

**
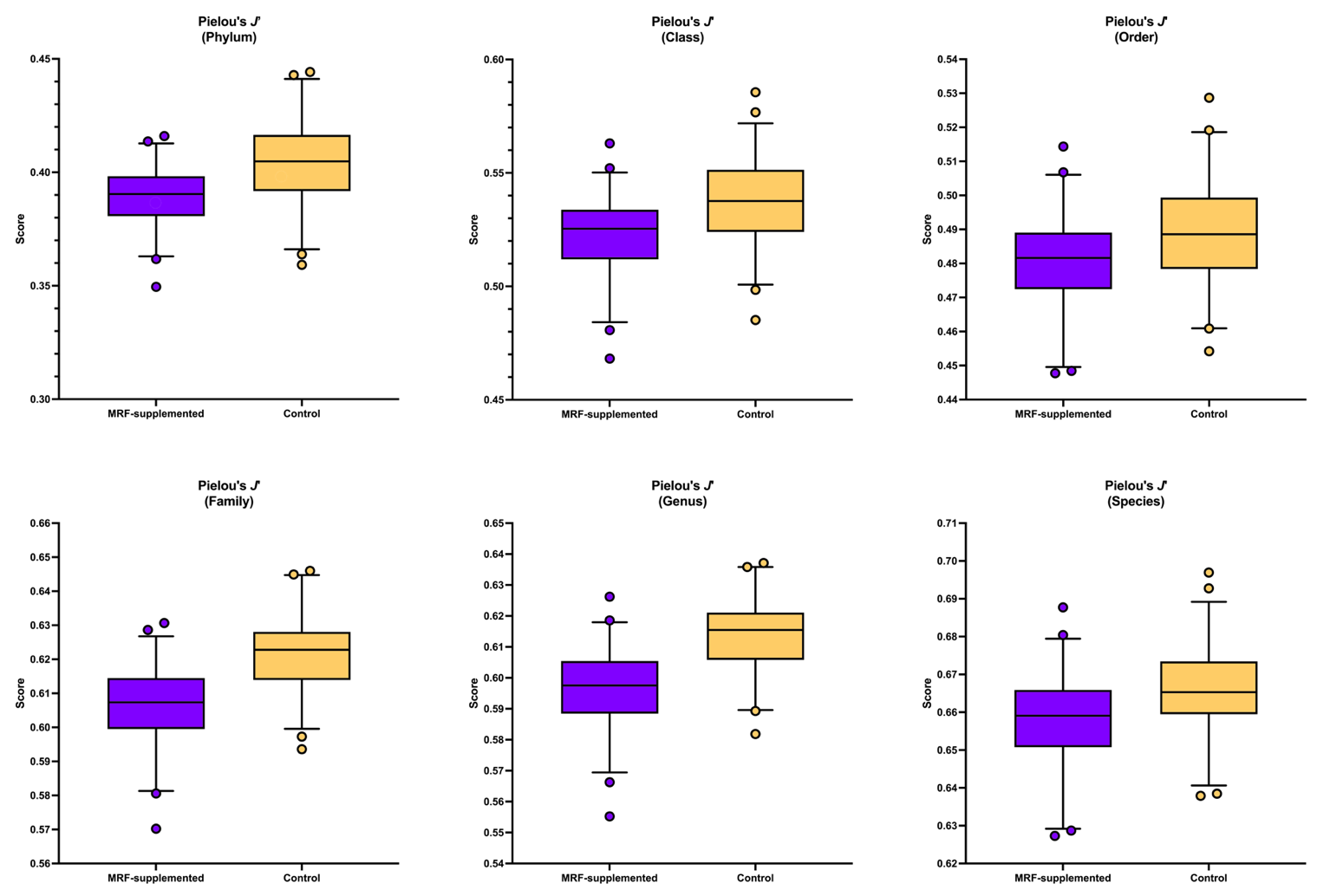
**

**SI Figure 4: Impact of MRF-supplementation on Pielou’s *J*’ (α-diversity)**

The horizontal line within each box denotes the mean and tails represent 95% confidence intervals. No observations exceeded the 95% CI intervals. In each plot *B* and *P* refer to the Brunner-Munzel test statistic and its associated *P*-value.

**
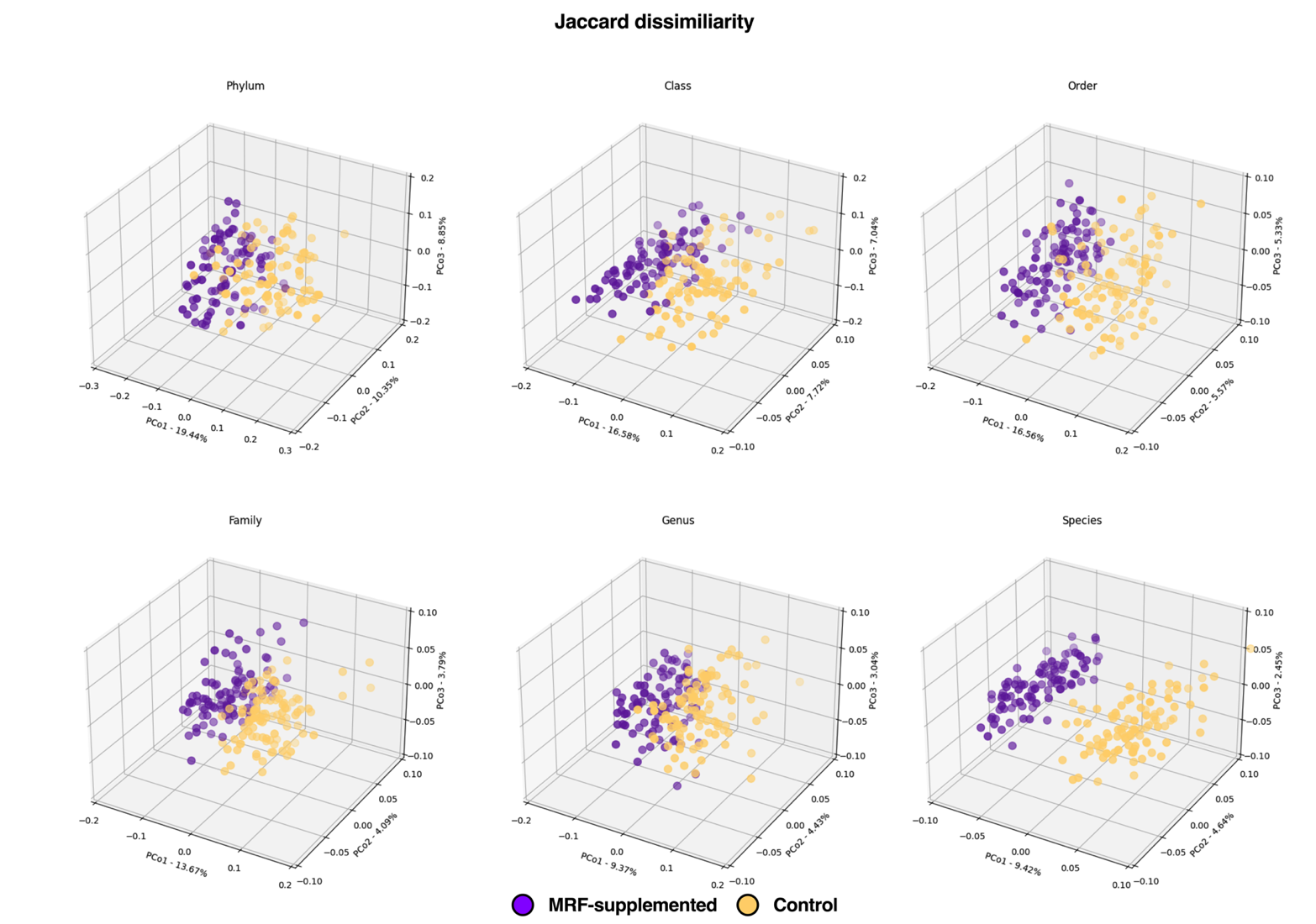
**

**SI Figure 5: Impact of MRF-supplementation on Jaccard dissimilarity (PCoA; β-diversity)**

Regularly spaced values represented on the *x*, *y*, and *z* axes are distance intervals as defined by their respective dissimilarity indices. The Principal Coordinates (PCo) for each axis are accompanied by their respective explained variances.

**
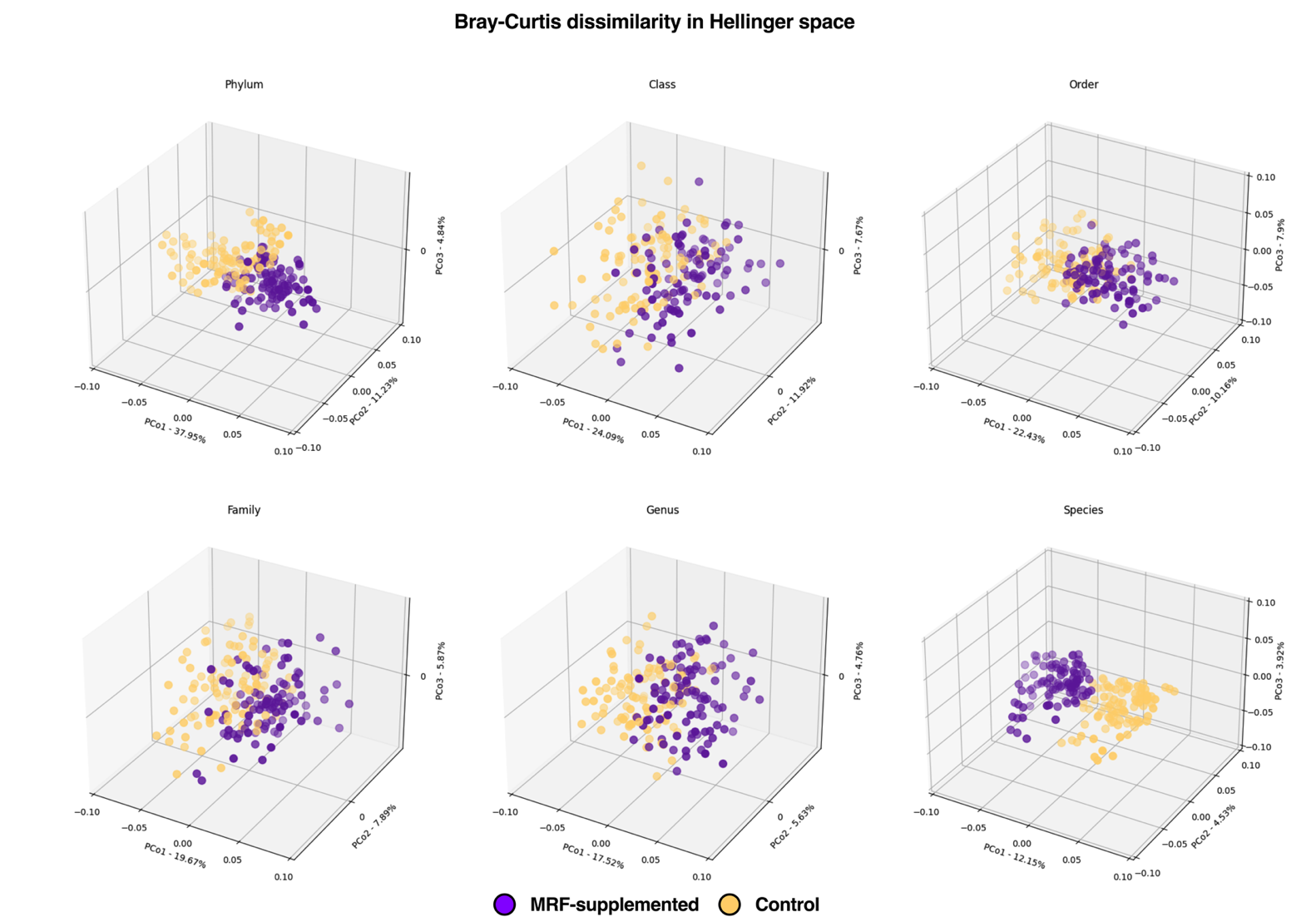
**

**SI Figure 6: Impact of MRF-supplementation on Bray-Curtis dissimilarity in Hellinger space (PCoA; β-diversity)**

Regularly spaced values represented on the *x*, *y*, and *z* axes are distance intervals as defined by their respective dissimilarity indices. The Principal Coordinates (PCo) for each axis are accompanied by their respective explained variances.

**
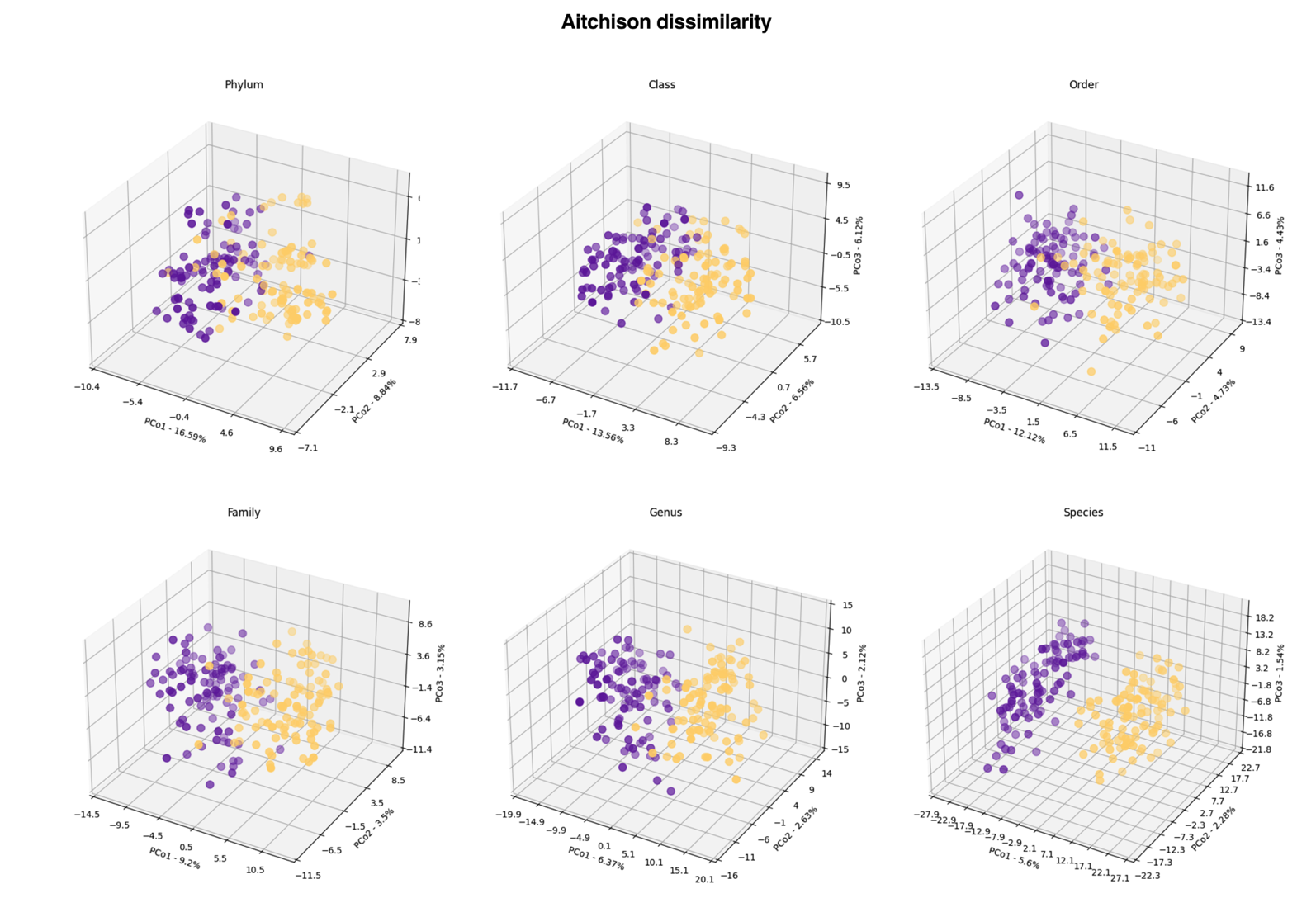
**

**SI Figure 7: Impact of MRF-supplementation on Aitchison dissimilarity (PCoA; β-diversity)**

Regularly spaced values represented on the *x*, *y*, and *z* axes are distance intervals as defined by their respective dissimilarity indices. The Principal Coordinates (PCo) for each axis are accompanied by their respective explained variances.

**
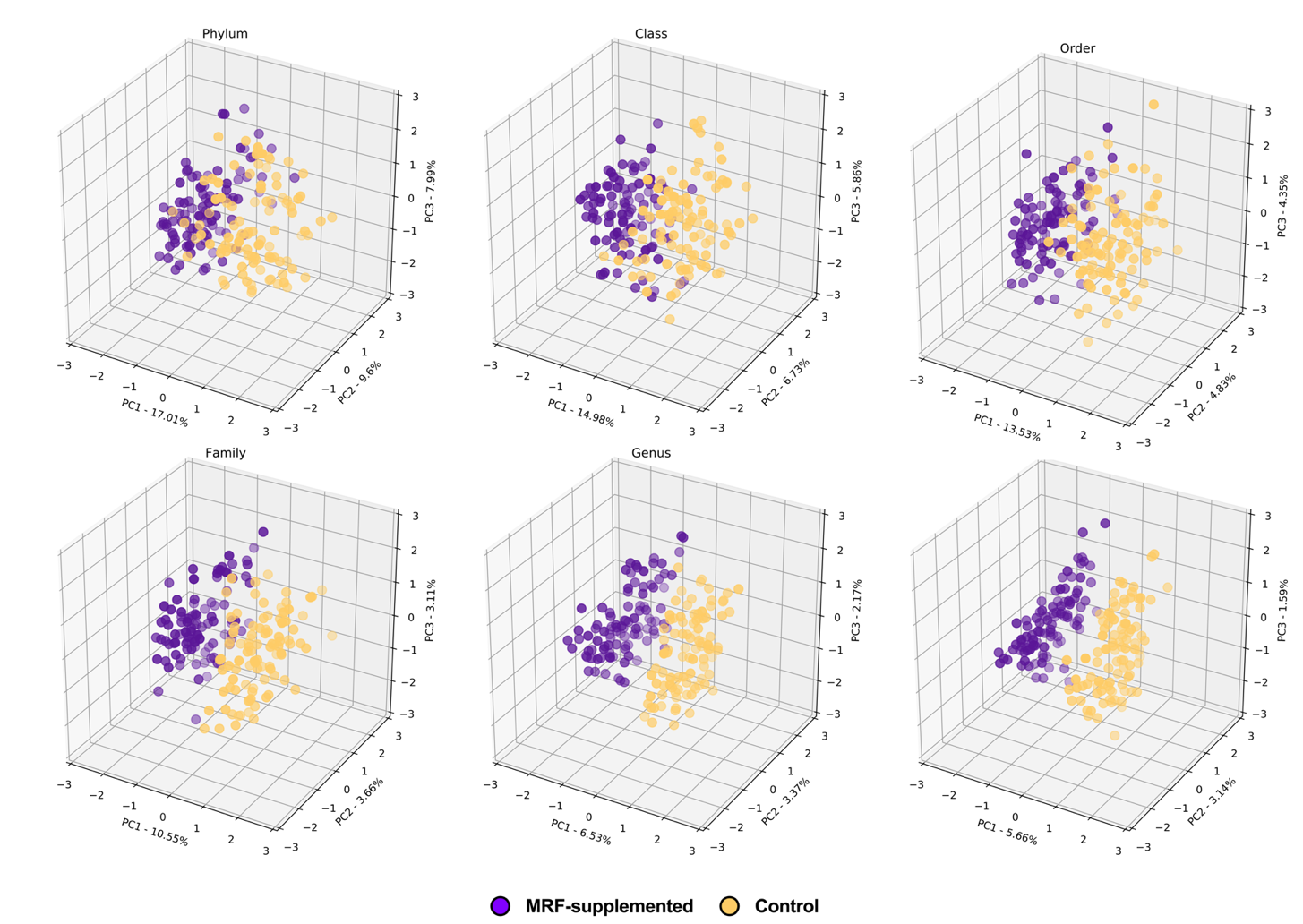
**

**SI Figure 8: Impact of MRF-supplementation on separability (PCA)**

Regularly spaced values represented on the *x*, *y*, and *z* axes are standard deviations away from the mean (0) in standardized (*Z*-score) space. The Principal Components (PC) for each axis are accompanied by their respective explained variances.
